# Inhibition of *Trpv4* rescues circuit and social deficits unmasked by acute inflammatory response in a Shank3 mouse model of Autism

**DOI:** 10.1038/s41380-021-01427-0

**Published:** 2022-01-12

**Authors:** Stamatina Tzanoulinou, Stefano Musardo, Alessandro Contestabile, Sebastiano Bariselli, Giulia Casarotto, Elia Magrinelli, Yong-hui Jiang, Denis Jabaudon, Camilla Bellone

**Affiliations:** 1 Department of Fundamental Neuroscience, CMU, University of Geneva, Geneva, Switzerland; 2 Department of Genetics, Yale University School of Medicine, New Haven, CT 06520 USA; 3 Present Address: Department of Biomedical Sciences (DSB), FBM, University of Lausanne, Lausanne, Switzerland

**Keywords:** Neuroscience, Autism spectrum disorders

## Abstract

Mutations in the *SHANK3* gene have been recognized as a genetic risk factor for Autism Spectrum Disorder (ASD), a neurodevelopmental disease characterized by social deficits and repetitive behaviors. While heterozygous *SHANK3* mutations are usually the types of mutations associated with idiopathic autism in patients, heterozygous deletion of *Shank3* gene in mice does not commonly induce ASD-related behavioral deficit. Here, we used in-vivo and ex-vivo approaches to demonstrate that region-specific neonatal downregulation of *Shank3* in the Nucleus Accumbens promotes D1R-medium spiny neurons (D1R-MSNs) hyperexcitability and upregulates Transient Receptor Potential Vanilloid 4 (*Trpv4)* to impair social behavior. Interestingly, genetically vulnerable *Shank3*^*+/−*^ mice, when challenged with Lipopolysaccharide to induce an acute inflammatory response, showed similar circuit and behavioral alterations that were rescued by acute Trpv4 inhibition. Altogether our data demonstrate shared molecular and circuit mechanisms between ASD-relevant genetic alterations and environmental insults, which ultimately lead to sociability dysfunctions.

## Introduction

Autism spectrum disorder (ASD) includes a heterogeneous group of neurodevelopmental diseases characterized by social communication deficits and repetitive behaviors. Mutations in *SHANK3* gene, coding for a scaffolding protein located at excitatory synapses, account for 1−2% of all ASD cases, and its haplo-insufficiency is acknowledged to lead to a high-penetrance form of ASD, known as Phelan-McDermid syndrome (PMS) [1, 2]. Currently, the development of pharmacological interventions to alleviate ASD-related sociability symptoms is limited by several factors, including the relative lack of understanding of the genetic consequences of *SHANK3* insufficiency. This is further complicated by the fact that Shank3 plays specific roles depending on its expression pattern in different regions and cell types [3]. Thus, investigating altered neuronal circuit mechanisms underlying disease pathophysiology and uncovering their roles in discrete behavioral readouts in mice is of the highest importance [4, 5].

Most of the pre-clinical models of *Shank3* deficiency show impairments in dorsal striatal circuits [3, 6], principally related to the indirect pathway, which drives repetitive behavior [6, 7]. On the other hand, the role of the mesolimbic reward system, including the Ventral Tegmental Area [8] and the Nucleus Accumbens (NAc) [9], in social reward processing makes it an ideal neural circuit substrate for further investigation in the context of ASD in both rodents [10] and humans [11, 12]. Despite the fact that neuronal deficits within the reward system have been revealed in different *Shank3* animal models [13, 14] and that expression of Shank3 in the striatum is particularly enriched [15], the contribution of *Shank3* insufficiency in the ventral striatum, which includes the NAc, to ASD symptomatology has been largely neglected.

Although the generation of knock-out (KO) *Shank3* (*Shank3*^*−/−*^) mouse lines has favored the identification of behavioral and synaptic impairments, single allele mutations minimally affect the behavioral pattern in rodents [14, 16–18] limiting translation from rodents to human studies. Indeed, while PMS patients are heterozygous for *SHANK3* deletions or mutations, most of the existing animal models failed to report consistent behavioral phenotypes when heterozygous mice were assessed (but see for example Lee et al. [19]). Thus, one intriguing question that arises is whether environmental challenges would exacerbate or unmask alterations, otherwise covert in heterozygous mice. Indeed, apart from genetic risk factors, several studies support the role of immune regulation and inflammation in ASD. Patients have frequent immune dysfunctions and immune-mediated co-morbidities [20]. Furthermore, transcriptomic analysis in post-mortem brain tissues revealed an upregulation of genes involved in inflammation [21], while in recent years, an interplay between immune system and reward circuit function has been put forward [22, 23]. Remarkably, in some PMS patients, debilitating symptoms appeared after acute infections or stressful environmental challenges [24] suggesting that the heterogeneity in the phenotypes could be the consequence of the interplay between genetic and environmental factors. Although increasing evidence indicates links between immune deficits and ASD, mechanistic insights are still lacking.

Here we firstly interrogated behavioral and electrophysiological consequences of shRNA-induced *Shank3* early postnatal downregulation in the NAc. Not only did we observe reduced social preference and D1R-medium spiny neurons (D1R-MSNs) hyperexcitability, but also identified the Transient Receptor Potential Vanilloid 4 (Trpv4) as a key effector of our observations. Remarkably, similar molecular, circuit, and behavioral alterations were also observed in genetically vulnerable *Shank3*^*+/−*^ mice challenged with lipopolysaccharide-induced neuroinflammation. Finally, acute Trpv4 inhibition in the NAc restored excitability and sociability deficits in *Shank3* heterozygous mice.

## Results

### Social deficits following early NAc-specific *Shank3* insufficiency

**Fig. 1:**
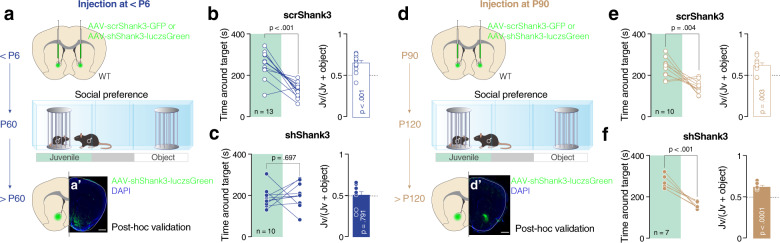
Downregulation of *Shank3* in the NAc during early postnatal development alters social preference. Schema of injection sites in the NAc with AAV-scr*Shank3*-GFP or AAV-sh*Shank3*-luczsGreen in P6 mice (**a**) or at P90 (**d**). **a′**, **d′** Representative images of injection sites (scale bar: 500 µm). Left: time spent around the enclosures during the social preference test for mice injected at P6 (**b** and **c**) or at P90 (**e** and **f**) (paired-samples t-tests for object- vs. social: **b** *t*_(12)_ = 6.092, *p* < 0.001; **c** *t*_(9)_ = 0.409, *p* = 0.697; **e** *t*_(9)_ = 3.806, *p* = 0.004; **f** *t*_(6)_ = 6.970, *p* < 0.001). Right: juvenile preference index for mice injected at P6 (**b** and **c**) or at P90 (**e** and **f**) (one-sample t-tests against chance level = 0.5: **b** *t*_(12)_ = 5.847, *p* < 0.001; **c** *t*_(9)_ = 0.273, *p* = 0.791; **e** *t*_(9)_ = 3.928, *p* = 0.003; **f** *t*_(6)_ = 7.996, *p* < 0.001). Error bars report SEM.

Given the emerging importance of NAc in social reward processing [9, 25], we first focused our investigation on this brain circuit and asked whether *Shank3* downregulation restricted to this region would lead to sociability deficits. Using AAV-sh*Shank3*-luczsGreen virus, we downregulated the expression of *Shank3* during early postnatal development [13, 26] (≤P6; hereafter P6 for simplicity, Fig. 1a-a′) or during adulthood (P90, Fig. 1d-d′). Both qPCR and Western blot analysis confirmed a decrease in *Shank3* gene and protein expression in the NAc (Supplementary Fig. 1a−d). To prove the specificity of the manipulation, western blot analysis of dorsal striatum did not reveal differences between scr*Shank3* (injected at the same age with a scrambled virus) and sh*Shank3* injected mice (Supplementary Fig. 1e).

While scr*Shank3* mice showed preference for a juvenile conspecific (Fig. 1b, e), sh*Shank3* P6-injected mice spent a comparable amount of time around the juvenile and object stimulus (Fig. 1c) and in the two chambers (Supplementary Fig. 2a). Furthermore, while the overall exploratory behavior was comparable between scr*Shank3* and sh*Shank3* mice (Supplementary Fig. 2b), sh*Shank3*-infected mice spent less time exploring the juvenile mouse and more time exploring the object stimulus compared to scr*Shank3* (Supplementary Fig. 2d, e). Remarkably, when shShank3 was injected at P90, mice showed intact sociability (Fig. 1f and Supplementary Fig. 2f) while presenting a similar decrease in Shank3 expression (Supplementary Fig. 1b). No difference in the exploration time around targets between groups (Supplementary Fig. 2g–i), nor in the distance moved (Supplementary Fig. 2j) was observed after injection at P90. No differences in the preference for unfamiliar mice in the social novelty paradigm (Supplementary Fig. 2k–n), nor the time spent in the open arms during the O-maze test (Supplementary Fig. 2o, p) were observed.

Altogether these data point at the NAc as a key region for sociability deficits induced by *Shank3* insufficiency. Moreover, our results suggest the existence of a critical period during early postnatal development, which is important for the expression of appropriate sociability later in life. We, thus, decided to focus our efforts on early *Shank3* downregulation and to investigate the mechanisms underlying sociability deficits.

### Alterations in intrinsic properties of NAc D1R-expressing medium spiny neurons following *Shank3* downregulation

**Fig. 2:**
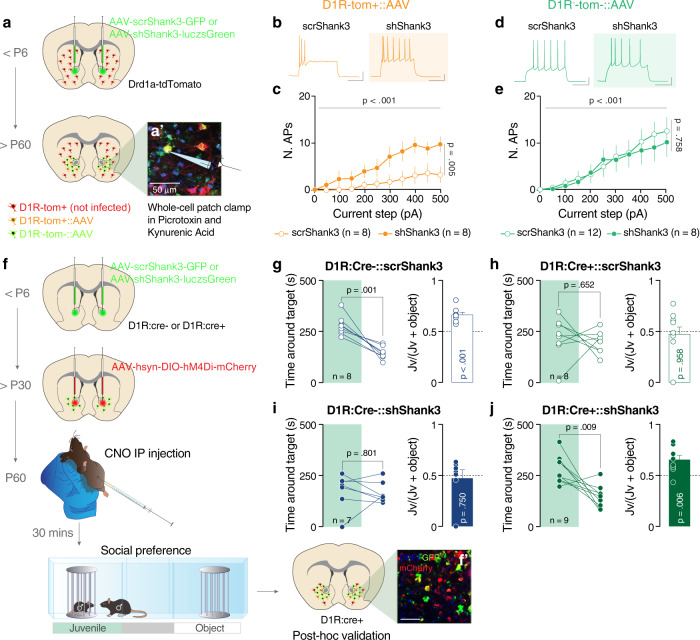
*Shank3* NAc downregulation alters D1R MSNs excitability. Decreasing the activity of D1R MSNs normalizes sociability deficits. **a** Experimental design. Drd1a-dTomato mice were injected neonatally in the NAc with scr or sh*Shank3* virus and whole-cell patch clamp recordings were performed during early adulthood. **a′** Representative image of the NAc of a D1R-tom+::sh*Shank3* mouse (scale bar: 50 µm). **b** Example traces at 300 pA depolarizing current injection in D1R-tom+ MSNs infected with scr*Shank3* (left) or with sh*Shank3* (right). **c** Number of action potentials (nAPs) across increasing depolarizing current steps (0-500 pA) for D1R-tom+::scr*Shank3* and sh*Shank3* MSNs, in presence of picrotoxin and kynurenic acid (repeated measures two-way ANOVA, virus main effect *F*_*(1, 14)*_ *= 10.88, p* = 0.005, current steps main effect *F*_*(10, 140)*_ *= 7.727, p* < 0.001, virus x current step interaction *F*_*(10, 140)*_ *= 1.626, p* = 0.1051, scr*Shank3* *n* = 8 cells, 3 mice, sh*Shank3* *n* = 8 cells, 3 mice). **d** Example traces at 300 pA depolarizing current injection in D1R-tom- MSNs infected with scr*Shank3* (left) or with sh*Shank3* (right). **e** Number of action potentials (nAPs) across increasing depolarizing current steps (0−500 pA) for D1R-tom−::scr*Shank3* and sh*Shank3* MSNs, in presence of picrotoxin and kynurenic acid (repeated measures (RM) two-way ANOVA, virus main effect *F*_*(1,18)*_ *=* 0.098, *p* = 0.758, current steps main effect *F*_*(10, 180)*_ *=* 14.58, *p* < 0.001, virus x current step interaction *F*_*(10, 180)*_ *= 0.3254, p* = 0. 9736, *n* = 8 cells, 3 mice (sh), *n* = 12 cells, 3 mice (scr)). **f** Experimental design. D1R-Cre positive (D1R:Cre^+^) and negative (D1R:Cre^−^) mice were injected neonatally in the NAc with scr or sh*Shank3* virus and after P30 with AAV-hSyn-DIO-hM4Di-mCherry (DREADD). After 4 weeks, allowing for virus expression, the mice underwent social behavior assessment in the three-chamber task. All mice were intraperitoneally injected with CNO 30 min before starting the test. **f′** Representative image of the NAc of a D1R:Cre^+^ mouse infected with sh*Shank3* and DREADD viruses (scale bar: 50 µm). Left: time around the target during the social preference test for D1R:Cre^-^::scr*Shank3* mice: paired-samples t-test for object- vs. social: **g** *t*_(7)_ = 5.453, *p* = 0.001; D1R:Cre^+^::scr*Shank3* mice, paired-samples t-test for object- vs. social: **h** *t*_(7)_ = 0.471, *p* = 0.652; D1R:Cre^−^::sh*Shank3* mice, paired-samples t-test for object- vs. social: **i** *t*_(6)_ = 0.264, *p* = 0.801 and D1R:Cre^+^::sh*Shank3* mice, paired-samples t-test for object- vs. social: **j** *t*_(8)_ = 3.443, *p* = 0.009. Right: juvenile preference index (one-sample t-tests against chance level = 0.5: D1R:Cre^−^::scr*Shank3*; *t*_(7)_ = 6.395, *p* < 0.001, D1R:Cre^+^::scr*Shank3*; *t*_(7)_ = 0.054, *p* = 0.958, D1R:Cre^−^::sh*Shank3*; *t*_(6)_ = 0.334, *p* = 0.750, D1R:Cre^+^::sh*Shank3*; *t*_(8)_ = 3.706, *p* = 0.006). Error bars report SEM.

The downregulation of Shank3 from D1R-expressing (direct pathway) or D2R-expressing MSNs of dorsal striatum (indirect pathway) leads to neuronal hyperexcitability [3]. To assess the excitability of MSN subpopulations in the NAc in our model, we used fluorescently-labeled D1R mice (Drd1a-tdTomato) [27] injected with scr*Shank3* or sh*Shank3* (Fig. 2a-a′). When recorded in presence of synaptic blockers (picrotoxin and kynurenic acid), *Shank3* downregulation increased the excitability of D1R-tom^+^ compared to scr*Shank3*::D1R-tom^+^ MSNs, while no changes were detected in the D1R-tom^−^ population (Fig. 2b–e and Supplementary Fig. 3a–f). Interestingly, in absence of synaptic blockers, *Shank3* downregulation induced a hyperexcitability of D1R-tom^+^ MSNs and hypo-excitability of D1R-tom^−^ MSNs, (Supplementary Fig. 3g–r). Overall, these results indicate that the hyperexcitability of accumbal direct pathway MSNs largely derives from alterations of intrinsic membrane properties, while the hypo-excitability of putative indirect pathway MSNs is the consequence of circuit network dysfunctions.

Shank3 down regulation has been previously associated with changes in the expression of glutamatergic receptors. We, therefore, assessed whether sh*Shank3* positive D1R MSNs presented deficits in the glutamatergic transmission onto MSNs within the NAc. Excitatory responses evoked by electrical stimulation revealed no difference in AMPA/NMDA ratio, rectification index, and paired-pulse ratio (PPR) between non-infected D1R positive cells and D1R cells infected with sh*Shank3* (Supplementary Fig. 3s−v).

### A causal link between NAc D1R MSN hyperexcitability and sociability deficits

To probe causality between direct pathway hyperexcitability and sociability deficits, we used cell-specific chemogenetic tools to manipulate neuronal activity. D1R:Cre^+^ or D1R:Cre^−^ mice were injected with either control scrambled virus or sh*Shank3* during early postnatal development. After P30, all mice were infected with an inhibitory Cre-dependent DREADD-expressing virus (AAV-DIO-hM4Di-mCherry) (Fig. 2f-f′). We validated the effectiveness of our chemogenetic approach by analyzing the expression of GIRK channels in NAc MSN (Supplementary Fig. 4a) the main effectors of chemogenetic inhibition, and the effects of Clozapine N-Oxide (CNO) on neuronal excitability ex-vivo (Supplementary Fig. 4b, c). Mice underwent the three-chamber interaction assay 30 min after systemic CNO injections (Fig. 2f-f′). D1R:Cre^+^ mice injected with CNO (regardless of NAc virus) reduced their locomotor activity (Supplementary Fig. 4d); however, the total exploration time for both enclosures remained comparable to that of D1R:Cre^−^ mice (Supplementary Fig. 4e). By analyzing the time spent around either the juvenile or object target, we confirmed that control D1R:Cre^-^::scr*Shank*3 mice showed intact sociability (Fig. 2g and Supplementary Fig. 4f). While both D1R:Cre^+^::scr*Shank*3 mice treated with CNO and D1R:Cre^−^::sh*Shank*3 did not show a preference for the social over the object stimulus (Fig. 2h, i and Supplementary Fig. 4f), interestingly, D1R:Cre^+^::shShank3 showed a preference for the social stimulus compared to the object (Fig. 2j and Supplementary Fig. 4f).

These data establish a causal link between NAc D1R-MSN hyperexcitability and sociability defects in-vivo, and further suggest that decreasing direct pathway hyperexcitability might be a useful strategy to ameliorate social dysfunctions.

### Downregulation of *Shank3* induces a *Trpv4* upregulation

**Fig. 3:**
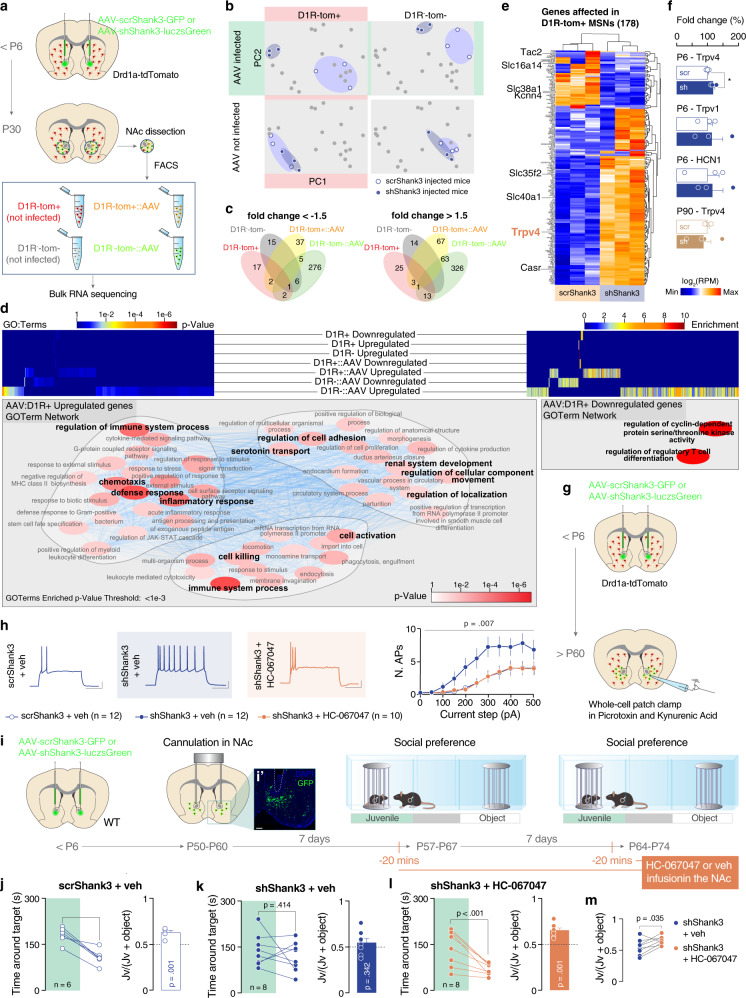
Downregulation of *Shank3* in D1R MSNs induces alterations in inflammatory mediators and *Trpv4* expression. **a** Experimental design. Drd1a-dTomato mice were injected neonatally in the NAc with scr or sh*Shank3* virus. At P30 the NAc was dissected and FACsorted in 4 different cell populations (D1R-tom+, D1R-tom-, D1R-tom+::AAV and D1R-tom−::AAV). For each cell population we carried out bulk RNA sequencing. **b** Worst-case scenario selected altered genes in scr vs sh testing clearly discriminated infected cells, both D1R+ and D1R− in PCA analysis. **c** While non-infected samples do not share common genes significantly altered in scr vs sh testing, infected D1R+ and D1R- share a core set of 68 altered genes. **d** Overall GO:Term analysis of infected D1R+ significantly altered genes highlights the relevance of inflammatory mechanisms, as well as cell adhesion-, localization- and movement-related functions. **e** D1R-tom+ altered genes include genes expressing proteins directly involved in electrophysiological properties, including the Transient receptor potential vanilloid 4 (*Trpv4*). **f** Real-time PCR analysis of NAc dissected from P6- or P90-injected mice confirm the upregulation of *Trpv4* in P6 sh-infected mice (unpaired t-tests: P6 – Trpv4 *t*_(4)_ = 2.980, *p* = 0.041; P6 – Trpv1 *t*_(4)_ = 0.367, *p* = 0.732; P6 – HCN1 *t*_(4)_ = 0.318, *p* = 0.766; P90 – Trpv4 *t*_(5)_ = 0.4203, *p* = 0.69). **g** Experimental design. Drd1a-dTomato mice were injected neonatally in the NAc with scr or sh*Shank3* virus and whole-cell patch clamp recordings were performed during early adulthood. **h** Right: example traces from 300 pA depolarizing current injection in D1R+ MSNs infected with scr*Shank3* treated with vehicle (left), D1R+ MSNs infected with sh*Shank3* treated with vehicle (middle) and D1R+ MSNs infected with sh*Shank3* treated with HC-067047 (right). Left: number of action potentials (nAPs) across increasing depolarizing current steps (0−500 pA) for D1R-tom+::scr*Shank3* and sh*Shank3* MSNs in the presence of Trpv4 antagonist (HC-067047) (repeated measures ANOVA, drug main effect *F*_*(2, 31)*_ *=* 5.883, *p* = 0.007, current steps main effect *F*_*(10, 310)*_ *=* 24.15, *p* < 0.001, drug by current steps interaction *F*_*(20, 310)*_ *=* 1.685, *p* = 0.035, *n* = 12 cells, 4 mice (sh*Shank3*-Veh), *n* = 10 cells, 3 mice (sh*Shank3*-Trpv4), *n* = 12 cells, 4 mice (scr*Shank3*-Veh)). **i** Experimental design. C57BL6/j mice were injected neonatally in the NAc with scr or sh*Shank3* virus and at P50-60 were bilaterally cannulated above the NAc. After 7 days, mice underwent the three-chamber social interaction assay. Scr*Shank3* were infused with vehicle (aCSF/DMSO 0.3%). On the other hand, sh*Shank3* mice were infused with either vehicle (aCSF/DMSO 0.3%) or HC-067047 (2 µg in aCSF/DMSO 0.3%) 10 min before to start the test. **i′** Representative image of the injection site and cannula placement above the NAc (scale bar: 250 µm). Left: time around the target during the social preference test for mice infected with scr*Shank3* and infused with vehicle (paired-samples t-test for object- vs. social: **j** *t*_(5)_ = 6.304, *p* = 0.002), mice infected with sh*Shank3* and infused with vehicle (paired-samples t-test for object- vs. social: **k** *t*_(7)_ = 0.869, *p* = 0.414) or with HC-067047 (paired-samples t-test for object- vs. social: **l** *t*_(7)_ = 4.324, *p* = 0.004). Right: juvenile preference index for mice infused either with vehicle or with HC-067047 (one-sample t-tests against chance level = 0.5: **j** *t*_(5)_ = 6.459, *p* = 0.001; **k** *t*_(7)_ = 1.02, *p* = 0.342; **l** *t*_(7)_ = 6.078, *p* = 0.001). **m** Juvenile preference index comparison between sh*Shank3*-vehicle and sh*Shank3*-HC-067047 (paired-samples t-test for object- vs. social: *t*_(7)_ = 2.6, *p* = 0.035). Error bars report SEM.

It has been previously shown that Shank3 mutations in humans predispose to autism by inducing a channelopathy [28]. To further investigate the mechanisms underlying D1R-MSN hyperexcitability, we performed direct pathway transcriptomic analysis of the NAc in Drd1a-tdTomato P6-injected scr*Shank*3 and sh*Shank*3 mice. For this purpose, we FAC-sorted direct pathway MSNs at P30 and performed bulk RNA sequencing (Fig. 3a). Scr*Shank3* and sh*Shank3* were clustered separately in both D1R-tom^+^ and D1R-tom^−^ populations (Fig. 3b) and differential expression analysis by groups for scr*Shank3 vs* sh*Shank3* revealed 178 altered genes in AAV-infected D1R-tom^+^ (Fig. 3c and Supplementary Fig. 5a). GO:Term analysis of significantly altered genes in NAc-injected shShank3 mice, highlighted changes relevant to cell adhesion, localization, and cellular movement-related, as well as, related to functions regarding inflammatory mechanisms (Fig. 3d). Moreover, within the modified genes identified in the bulk RNA sequencing, we observed a high representation of activity-related genes (Fig. 3e) in D1R-tom^+^ neurons, immune response-related genes, as well as, SFARI genes associated with ASD in both D1R-tom^+^ and D1R-tom^−^ populations (Supplementary Fig. 5b, c).

Among these genes altered by early postnatal *Shank3* downregulation, we noticed that the one encoding for the Transient receptor potential vanilloid 4 (*Trpv4*) channel was significantly upregulated (Fig. 3e). Trpv4 is a member of the transient receptor potential superfamily, broadly expressed in the central nervous system [29]. These receptors are activated by temperature, mechanical stimulation, cell swelling, and endocannabinoids [30] and participate in inflammatory responses [31]. Moreover, Trpv4 function influences neuronal excitability and its disruption leads to social behavior abnormalities [32]. The increase in Trpv4 expression in the NAc from mice where Shank3 was downregulated before P6, was confirmed by qPCR (Fig. 3f). To further consolidate the potential link between downregulation of *Shank3*, overexpression of *Trpv4*, and increased excitability, we evaluated the expression of *Trpv1* and *HCN1* genes which have been found as neuronal excitability regulators in other system [33–35]. RT-qPCR analysis showed that these genes are not altered after *Shank3* downregulation (Fig. 3f). Remarkably, when Shank3 was downregulated during adulthood, the levels of Trpv4 were comparable between scrShank3 and shShank3 -injected mice (Fig. 3f). Since downregulation of *Shank3* in adulthood did not reveal any sociability deficit (Fig. 1d−f), together, these data suggested a link between the increased *Trpv4* expression and the behavioral phenotype. To directly interrogate this hypothesis, we tested the ability of a Trpv4-specific inhibitor (HC-067047) to rescue the direct pathway MSN hyperexcitability ex-vivo (Fig. 3g). In patch-clamp recordings, bath application of HC-067047 normalized the excitability of D1R-tom^+^::sh*Shank3* to D1R-tom^+^::scr*Shank3* levels (Fig. 3h). So far, this evidence indicates that early downregulation of *Shank3* in the NAc upregulates both the expression and the function of *Trpv4* in the direct pathway neurons, identifying a novel molecular effector of *Shank3* insufficiency.

### Trpv4 antagonist restores sociability in NAc sh*Shank3* mice

To test causality between sociability defects and the upregulation of *Trpv4* in the NAc and to probe its potential as a therapeutic target in-vivo, we next asked whether the region-specific administration of Trpv4 inhibitor restores sociability in sh*Shank3* mice. Scr*Shank3* and sh*Shank3* were bilaterally cannulated above the NAc for local pharmacology experiments. After one week of recovery, sh*Shank3* mice were pre-treated with HC-067047 or vehicle before the three-chamber test (Fig. 3i-i**′**). Treatments were counterbalanced and the same animals were tested again after seven days (scr*Shank3* animals were instead infused only with vehicle). Scr*Shank3* mice infused with vehicle showed intact sociability (Fig. 3j) indicating no side effects of the cannulation on our behavioral endpoints. Confirming our previous findings, vehicle-infused sh*Shank3* animals showed impaired social preference (Fig. 3k). Remarkably, intra-NAc Trpv4 antagonist (HC-067047) infusions in sh*Shank3* mice restored sociability (Fig. 3l), increasing the time spent in the social chamber (Supplementary Fig. 5d). Furthermore, sh*Shank3* mice showed an increase of social preference ratio when infused with HC-067047 compared to when infused with vehicle (Fig. 3m). No difference was observed in the distance moved during the test among the groups (Supplementary Fig. 5e).

Our results highlight the role of Trpv4 both in D1R-MSN hyperexcitability and social preference deficits displayed by NAc-injected sh*Shank3* mice.

### LPS challenge unmasks social deficits in *Shank3*^*+/−*^ mice

**Fig. 4:**
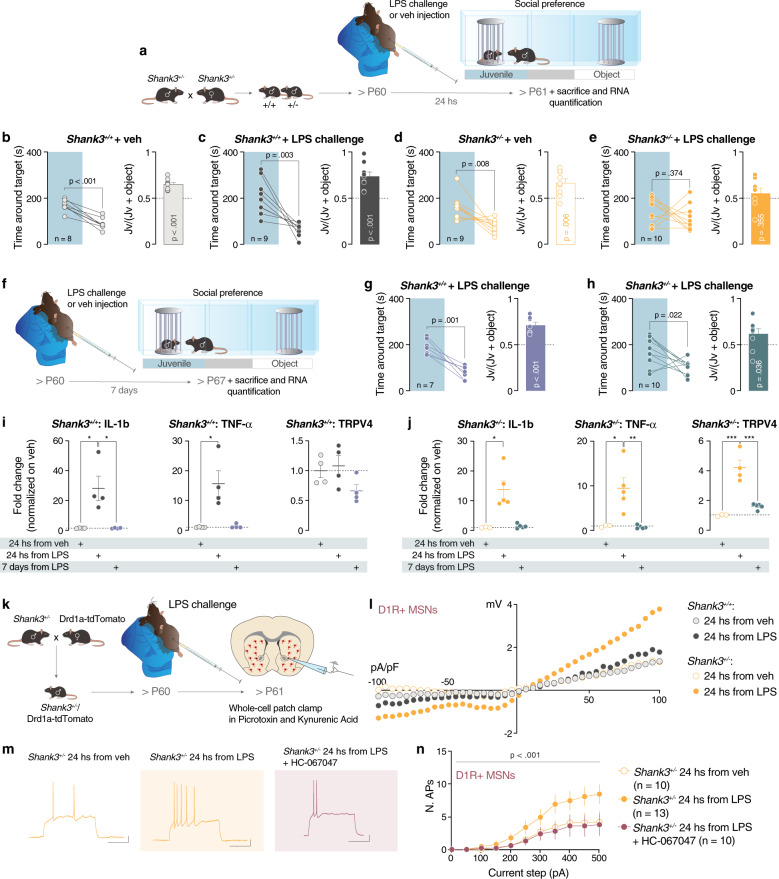
LPS challenge in *Shank3*^*+/−*^ unmasks social deficits. **a** Experimental design. *Shank3*^*+/+*^ and *Shank3*^*+/−*^ were intraperitoneally injected with LPS or vehicle and 24 h later they were subjected to the three-chamber task. Left: Time spent around the target (paired-samples t-tests for object- vs. social: **b** *t*_(7)_ = 7.686, *p* < 0.001; **c** *t*_(8)_ = 4.199, *p* = 0.003; **d** *t*_(8)_ = 3.462, *p* = 0.008; **e** *t*_(9)_ = 0.935, *p* = 0.374). Right: juvenile preference index (one-sample t-tests against chance level = 0.5: **b** *t*_(7)_ = 7.2, *p* < 0.001; **c** *t*_(8)_ = 5.262, *p* < 0.001; **d** *t*_(8)_ = 3.734, *p* = 0.006; **e** *t*_(9)_ = 0.9747, *p* = 0.355). **f** Experimental design. *Shank3*^*+/+*^ and *Shank3*^*+/−*^ were intraperitoneally injected with LPS and 7 days later were subjected to a three-chamber task. Left: Time spent around the target (paired-samples t-tests for object- vs. social: **g** *t*_(6)_ = 5.979, *p* = 0.001; **h** *t*_(9)_ = 2.759, *p* = 0.022). Right: juvenile preference index (one-sample t-tests against chance level = 0.5: **g** *t*_(6)_ = 6.054, *p* < 0.001; **h** *t*_(9)_ = 2.463, *p* = 0.036). **i** mRNA expression analysis of *IL-1β*, *TNF-α* and *Trpv4* genes after LPS challenge in *Shank3*^*+/+*^ (*IL-1β* one-way ANOVA followed by Sidak’s multiple comparisons test, *F*_*(2, 9)*_ *= 10.33, p* = *0.005*; *TNF-α* Kruskal−Wallis statistic 7.538, *p* = *0.012; Trpv4* one way ANOVA followed by Sidak’s multiple comparisons test, *F*_*(2, 9)*_ *= 2.768, p* = *0.116*). **j** mRNA expression analysis of *IL-1β*, *TNF-α* and *Trpv4* genes after LPS challenge in *Shank3*^*+/−*^ (IL-1*β* Kruskal−Wallis statistic 9.002, *p* = *0.002*; TNF-α one way ANOVA followed by Sidak’s multiple comparisons test, *F*_*(2, 10)*_ *= 10.27, p* = *0.004; Trpv4* one way ANOVA followed by Sidak’s multiple comparisons test: *F*_*(2, 9)*_ *= 31.26, p* < *0.001*). **k** Experimental design. *Shank3*^*+/−*^ were crossed with Drd1a-tdTomato mice labeling specifically D1R-MSNs in a *Shank3*^*+/−*^ background. Ex-vivo patch clamp recordings were made 24 h after the LPS injection. **l** Whole-cell recording of Trpv4 current 24 h after LPS challenge in *Shank3*^*+/+*^ and *Shank3*^*+/−*^ mice (repeated measures ANOVA, voltage steps main effect *F*_*(1.171,25.77)*_ *=* 12.11, *p* = *0.001*, genotype main effect *F*_*(3, 22)*_ *=* 0.4152, *p* = *0.744*, genotype by voltage steps interaction *F*_*(120, 880)*_ *=* 1.451, *p* = 0.002; *n* = 5 cells, 2 mice (*Shank3*^*+/+*^), *n* = 5 cells, 2 mice (*Shank3*^*+/+*^ + LPS), *n* = 7 cells, 2 mice (*Shank3*^*−/+*^) *n* = 9 cells, 2 mice (*Shank3*^*−/+*^ + LPS)). **m** Example traces from 300 pA depolarizing current injection in D1R + MSNs of *Shank3*^*+/−*^ mice 24 h after: vehicle IP injection and treated with vehicle (left), LPS challenge and treated with vehicle (middle), LPS challenge and treated with HC-067047 (right). **n** Number of action potentials (nAPs) across increasing depolarizing current steps (0−500 pA) for D1R-tom+::*Shank3*^*+/−*^ MSNs after LPS challenge (repeated measures ANOVA, treatment main effect *F*_*(2, 30)*_ *=* 3.034, *p* = 0.063, current steps main effect *F*_*(10, 300)*_ *=* 28.08, *p* < 0.001, treatment by current steps interaction *F*_*(20, 300)*_ *=* 2.042, *p* = 0.006, *n* = 10 cells, 3−4 mice each group). Error bars report SEM.

Single allele mutations of *Shank3* minimally affect the behavioral pattern in rodents [14, 16–18]. For instance, *Shank3* heterozygous mutant mice, in which exons 4 to 22 were deleted (Δe4-22^*+/−*^ hereafter referred to as *Shank3*^*+/−*^) [18] showed mild decrease in Shank3 proteins expression (Supplementary Fig. 6a) and do not display social preference deficits in the three-chamber test (Supplementary Fig. 6b−f). The differences observed in the behavioral outcomes of the sh*Shank3* and Shank3 knock-out models used in this study could be explained by the different expression of Shank3 isoforms (Supplementary Fig. 6a) or by the different temporal downregulation of the protein. On the other hand, based on the region-specific results obtained by GO:Term analysis (Fig. 3d), we hypothesized that acute inflammatory challenge induced by LPS could unmask behavioral deficits of *Shank3*^*+/−*^ mice via a mechanism dependent on Trpv4. Remarkably, when LPS was injected 24 h prior the three-chamber task (Fig. 4a), *Shank3*^*+/−*^ mice spent a comparable amount of time around the juvenile and object stimulus and in the corresponding chamber, indicating sociability deficits (Fig. 4e and Supplementary Fig. 7a). As control, saline-injected *Shank3*^*+/+*^ and *Shank3*^*+/−*^ mice spent more time exploring the juvenile-containing enclosure and chamber (Fig. 4b, d and Supplementary Fig. 7a). Moreover, LPS challenge did not confer any behavioral alterations in *Shank3*^*+/+*^ mice (Fig. 4c and Supplementary Fig. 7a) and the distance moved did not differ across genotypes (Supplementary Fig. 7b). Importantly, sociability deficits were not observed 7 days after LPS injection (Fig. 4f−h and Supplementary Fig. 7c, d) indicating that alterations induced by acute inflammatory challenges were transient.

We next asked whether striatal *Trpv4* expression was altered in Shank3^*+/−*^ mice after LPS. Whereas LPS injections increased the inflammatory markers IL-1β and TNF-α expression 24 h after LPS injection in both *Shank3*^*+/+*^ and *Shank3*^*+/−*^ mice, the observed increase in *Trpv4* was only seen in *Shank3*^*+/−*^ mice and not detectable 7 days after LPS challenge (Fig. 4i, j). Interestingly, overexpression of Trpv4 induced by injection of AAV-hSyn-mTRPV4-2A-eGFP in the NAc during adulthood does not alter sociability in *Shank3*^*+/−*^ mice (Supplementary Fig. 8a−g). These data support the hypothesis that inflammatory challenges, but not the TRPV4 overexpression by itself, unmask behavioral phenotypes in *Shank3*^*+/−*^ mice.

### Hyperexcitability seen in D1R-MSNs of *Shank3*^*+/−*^ mice after an acute LPS challenge is rescued by Trpv4 antagonist ex-vivo

To further investigate our hypothesis, we crossed *Shank3*^*+/−*^ with Drd1a-tdTomato mice and we performed ex-vivo patch-clamp recordings from D1R-MSNs 24 h after LPS injection (Fig. 4k). In order to probe the functional consequences of *Trpv4* upregulation, we first assessed Trpv4-mediated whole-cell currents from D1R-MSNs and observed an increase only in LPS-challenged *Shank3*^*+/*−^ mice (Fig. 4l). Importantly, we found that similarly to the NAc-sh*Shank3* model, LPS challenge in *Shank3*^*+/−*^ mice caused hyperexcitability in D1R-MSNs and not in putative D2R-MSNs (Fig. 4m, n and Supplementary Fig. 8e). Furthermore, the excitability of D1R-MSNs in *Shank3*^*+/−*^ mice returns to normal levels 7 days after the LPS challenge (Supplementary Fig. 8f). Finally, bath application of Trpv4 antagonist, HC-067047, normalized neuronal excitability, strengthening the causal links between D1R-MSN hyperexcitability and *Trpv4* upregulation (Fig. 4m, n).

### Acute LPS challenge alters the calcium transients of NAc cells in *Shank3*^*+/−*^ mice during sociability test

**Fig. 5:**
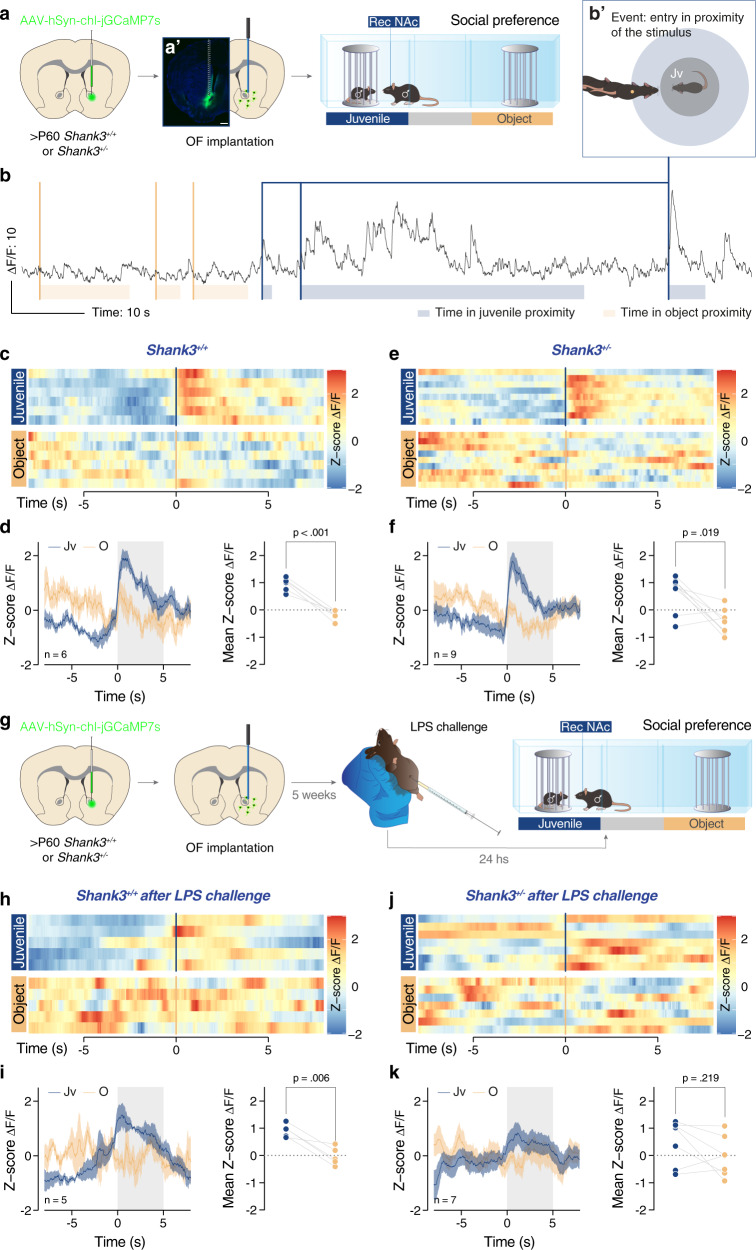
Acute LPS challenge alters the calcium transients of NAc cells in *Shank3*^*+/−*^ mice during sociability test. **a** Experimental design. Adult *Shank3*^*+/+*^ and *Shank3*^*+/−*^ mice were unilaterally injected in the NAc with an AAV-hSyn-chl-jGCaMP7s virus and subsequently an optic fiber was implanted above the ROI. These animals were then tested in the 3- chamber task. **a′** Representative image of injection site and optic fiber implantation (scale bar: 500 µm). **b** Example of recorded Δ*F*/*F* during the three-chamber task. The entry in proximity of the stimuli are indicated with bold lines (entry in the juvenile proximity in blue and orange for the object). The time passed in proximity of the stimuli is indicated below the traces. **b′** Schema reporting when the experimental animal is entering in proximity to the juvenile stimulus. **c, e, h**, and **j** Heatmaps reporting the PETH of normalized (z-score) Δ*F*/*F* recorded in the NAc and centered on the entry in proximity of the stimuli (time = 0). **d, f, i**, and **k** Left: PETH of normalized (z-score) Δ*F*/*F* recorded in the NAc and centered on the entry in proximity of the stimuli (time = 0). Right: Comparison of the juvenile (Jv, in blue) and object (O, in orange) mean z-scores obtained on averaging the normalized Δ*F*/*F* 5 s after the entry in proximity of the stimuli (paired-samples t-tests for object- vs. social: **d** *t*_(5)_ = 8.767, *p* < 0.001; **i** *t*_(4)_ = 5.301, *p* = 0.006. Wilcoxon test for object- vs. social: **f** *W* = −39.00, *p* = 0.019; **k** *W* = −16.00, *p* = 0.219). **g** Experimental design. After a pause of 7 days, the mice that performed the experiments described in (**a**) were intraperitoneally injected with LPS and 24 h later were subjected to a second three chambers test. Abbreviations: OF optic fiber, Jv juvenile, O object. Error bars report SEM.

To explain the link between neuronal excitability and behavioral deficits, we recorded in-vivo calcium transients using fiber photometry. Specifically, we unilaterally injected a GCamp7s-expressing virus (ssAAV5/2-hSyn1-chl-jGCamp7s-WPRE-SV40p(A)) in the NAc of *Shank3*^*+/+*^ and *Shank3*^*+/−*^ mice (Fig. 5a). We then implanted an optic fiber above the previously injected NAc to record somatic Ca^2+^ transients during the three chambers interaction task (Fig. 5a-a′). During this experiment, the central point of the experimental mouse was detected and tracked. Consequently, it was possible to assess the entry in the zone proximal to the stimuli and the time spent in this zone (Fig. 5b-b′). Peri-event time histogram (PETH) measured in naïve *Shank3*^*+/+*^ and *Shank3*^*+/−*^ mice revealed a significant increase in normalized ΔF/F (Z-score) for both genotypes immediately after the entry in proximity of the stimulus animal (normalized time = 0, Fig. 5c−f). On the other hand, the entry in proximity of the object did not elicit any increase in calcium transients and the mean z-score Δ*F*/*F* (mean of z-score Δ*F*/*F* in the time interval [0; 5]) was significantly different between the two stimuli in both groups of mice (Fig. 5c−f).

In a second phase, we repeated the experiment 24 h after challenging the same mice with LPS (Fig. 5g). Although the LPS challenge did not affect the activity of *Shank3*^*+/+*^ neurons in the NAc (Fig. 5h, i), *Shank3*^*+/−*^ mice did not show the peak of normalized Δ*F*/*F* upon entering in juvenile proximity and, indeed, presented a similar mean z-score Δ*F*/*F* between juvenile and object stimuli. Collectively, these data strongly suggest that the neuronal hyperexcitability induced by inflammatory challenge occludes the increase in calcium transient within the NAc during the sociability task. Our hypothesis was further supported by post hoc quantification, revealing an increased density of cFos positive neurons in the NAc 24 h after LPS injection (Supplementary Fig. 9a−c).

### Intra-NAc Trpv4 antagonist restores sociability in *Shank3*^*+/−*^ LPS-challenged mice

**Fig. 6:**
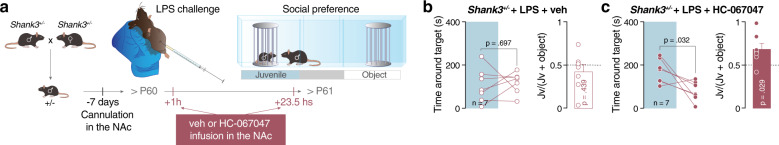
Trpv4 antagonist infused in the NAc improves social deficits in *Shank3*^*+/−*^ mice challenged with LPS. **a** Experimental design. Adult *Shank3*^*+/−*^ mice were intraperitoneally injected with LPS and 24 h later were subjected to the behavioral task. 30 min before the test, mice were infused (in the NAc) either with Trpv4 antagonist (HC-067047) or vehicle. Left: Time spent around the target for *Shank3*^*+/*^ mice after LPS challenge and vehicle or HC-067047 infusion in the NAc (paired-samples t-tests for object- vs. social: **b** *t*_(6)_ = 0.408, *p* = 0.697; **c** *t*_(6)_ = 2.787, *p* = 0.032). Right: juvenile preference index (one-sample t-tests against chance level = 0.5: **b** *t*_(6)_ = 0.629, *p* = 0.439; **c** *t*_(6)_ = 2.852, *p* = 0.029). Error bars report SEM.

To further investigate whether NAc plays a role in the behavioral alterations observed in *Shank3*^*+/−*^ mice after LPS injection, *Shank3*^*+/−*^ mice were bilaterally cannulated above the NAc for local pharmacology experiments aimed at inhibiting Trpv4 channels. After one week of recovery, mice were treated with either HC-067047 or vehicle intra-NAc infusions 1 h after the LPS challenge. The day after, mice were again infused locally in the NAc with HC-067047 or vehicle 30 min before the three-chamber task (Fig. 6a). While *Shank3*^*+/−*^::LPS mice infused with vehicle showed sociability deficits (Fig. 6b and Supplementary Fig. 10a), *Shank3*^*+/−*^::LPS mice infused with HC-067047 spent more time around the enclosure containing the juvenile mouse (Fig. 6c), albeit without a significant difference in the time spent in chambers (Supplementary Fig. 10a). Locomotor activity was not affected by local HC-067047 treatment (Supplementary Fig. 10b). These results indicate that the inhibition of *Trpv4* in the NAc after immune system activation is sufficient to ameliorate sociability deficits, suggesting a link between *Trpv4* modulation and social behavior.

Collectively, our data highlight the NAc *Trpv4* alterations as a potentially common and unifying molecular underlying factor in sociability and aberrant intrinsic neuronal properties in *Shank3* mouse models for autism.

## Discussion

Mutations in the *SHANK3* gene have been recognized as a genetic risk factor for ASD. Remarkably, high heterogeneity of neuronal pathophysiology and behavioral phenotypes have been reported in *Shank3* mouse models. Nevertheless, whether environmental factors contribute to the phenotypic heterogeneity of *Shank3* mouse model is still largely unknown. Here we first found that early loss of *Shank3* in the NAc reduces sociability via direct pathway hyperexcitability. These changes were accompanied by an unbalance of inflammatory mediators and by the overexpression of *Trpv4*. Interestingly, lipopolysaccharide-induced neuroinflammation revealed similar molecular, circuit, and behavioral alterations in genetically vulnerable *Shank3*^+/*−*^ mice. Acute Trpv4 inhibition in the NAc restored excitability and sociability deficits. Our data not only suggest that activation of the immune system may unmask autism-related behavioral phenotypes in genetically vulnerable mice but also ascribe Trpv4 as a potential therapeutic target for sociability defects in Autism.

The mesolimbic system represents an interesting hub for ASD pathophysiology. Indeed, human studies reported that social stimuli activate the NAc [36–40] and that this activation is disrupted in ASD patients [11, 41]. In support of the clinical studies, alterations in the mesolimbic system induce reward-related behavioral alterations in rodents [8, 13, 42]. However, the neuronal mechanisms underlying NAc-related sociability deficits remained largely unknown. It has been previously shown that the lack of *Shank3* induces differential alterations of intrinsic and synaptic properties of dorsolateral striatum D1R- and D2R-MSNs and that deficits in the indirect pathway contribute to repetitive behavior [3, 6]. In our study, we found that the downregulation of *Shank3* in the ventral striatum alters sociability via hyperexcitability of D1R-MSNs. While we cannot exclude that changes in D2R-MSNs also contribute to the phenotype, it is important to note that changes in excitability in the indirect pathway neurons were only observed in absence of synaptic blockers. Furthermore, while decreasing the activity of D1R-MSNs in sh*Shank3* mice was able to rescue the behavioral phenotype, decreasing the activity of the direct pathway neurons in control mice alters sociability (Fig. 2h). These findings strongly suggest that too low or too high D1R-MSNs activity affects sociability and are in line with previous evidence supporting the importance of NAc D1R-MSNs activity in modulating social behavior [25]. It should be noted, however, that while it has been shown that acute direct stimulation of NAc D1R-MSNs increases social interaction [25], the chronic increase in excitability during postnatal development in shShank3 mice alters sociability probably as a consequence of altered expression of several genes. Overall, our data not only causally link the activity of the direct pathway ventral striatum to sociability but suggest that the activity of D1R-MSN has to be tightly tuned in order to guarantee the optimal expression of social behavior.

During development synaptic connections and brain circuits change in response to experiences and surrounding environments. These changes occur during specific time windows called critical periods [43], which represent not only an opportunity for the developing brain, but also vulnerable period more sensitive to negative experience and structural alterations. Understanding the impact of the critical period in the context of ASD pathogenesis is still an open question. Previously it has been shown that post-development activation of *Shank3* rescues selective autistic-like phenotype in an ASD mouse model, indicating a certain degree of continued plasticity in adult diseased brain [16]. Here we prove that NAc downregulation of *Shank3* early in life, but not in adulthood, induces sociability deficits emphasizing again how changes that occur during development may have a different impact when circuits are established.

Although ASD is defined as a synaptic pathology [44, 45], recent evidence demonstrated a fundamental role of ion channels deficits in the pathophysiology of ASDs. Indeed, the loss of scaffolding between Shank3 and HCN impairs Ih currents and neuronal excitability [28]. Given that our electrophysiological recordings indicated an increased excitability of D1R-MSNs when *Shank3* was downregulated, we focused our attention on genes whose expression is linked to neuronal activity. It should be noted that we do not exclude that other genes encoding for ion channels could contribute to the observed changes in excitability. Nevertheless, here we highlight a novel link between Trpv4 alterations and Shank3 insufficiency. Specifically, we found that accumbal *Shank3* insufficiency upregulates *Trpv4*, a non-selective cation channel constitutively active at physiological temperatures [46], which allows Ca^2+^ influx, stimulates Ca^2+^-induced Ca^2+^-release (CICR) signaling [47–49], and ultimately tunes neuronal excitability [46]. Moreover, we have shown that inhibiting Trpv4 was sufficient to restore excitability and behavioral phenotype strengthening the link between Trpv4 and Shank3. Interestingly, we have not observed an upregulation of *Trpv4* in P90-injected sh*Shank3* mice. To further prove the causal link between the gene and behavior, we observed an increase in Trpv4 expression in *Shank3*^*+/−*^ mice 24 h after LPS, timepoint at which we also could observe behavioral deficits. Furthermore, sociability of sh*Shank3* and LPS-*Shank3*^*+/−*^ mice improves by inhibiting Trpv4 in a region-specific manner. Although future experiments will have to determine the precise mechanisms of how a scaffold protein could affect the transcription of a set of genes, our study supports the idea that *Shank3* downregulation affects both intrinsic excitability and synaptic properties, which may ultimately account for the symptom heterogeneity of PMS patients.

According to previous research, *Shank3* downregulation in the VTA early in life also leads to sociability deficits, albeit of different nature [13]. Indeed, VTA-sh*Shank3* mice show a specific deficit in maintaining interest for social approach, while NAc-sh*Shank3* mice showed reduced sociability throughout the test duration. The NAc is a key region of the mesolimbic dopamine circuit, being the main target of VTA DA neurons and DA release in the NAc plays an important role in motivated behavior. Interestingly, the impact of DA manipulation on motivated behavior is highly complex and, although our data suggest a different contribution of the NAc and VTA in social motivation, further studies will need to disentangle their precise role.

The heterogeneity of ASD symptoms most likely results from the involvement of a multitude of genetic factors and a complex interaction between those genes and environmental challenges [50–55]. For example, increasing evidence suggests a role for inflammation in ASD pathogenesis [56–58]. Indeed, individuals with ASD often have heightened levels of pro-inflammatory cytokines [59, 60], and post mortem brain samples revealed an upregulation of genes related to the immune response [61]. A recent hypothesis posits that the activation of the immune system during critical periods of brain development may cause neuronal dysfunctions [62, 63] and lead to behavioral deficits [64]. To better understand how immune responses to infectious agents might affect behavior in preclinical models, we used LPS, a bacterial endotoxin, that stimulates an innate response to bacterial infection leading to a variety of behavioral changes [65–70]. Interestingly, animals exposed to inflammatory stimuli show impaired motivation, decreased exploratory behavior [71], and social withdrawal [72, 73]. Here, we show that one LPS injection during adulthood reveals transient sociability deficits in adult *Shank3*^*+/−*^ mice. Although the LPS challenge used in our study was acute and the reported in-vivo and ex-vivo deficits were ameliorated after one week, future studies would need to examine whether a chronic immune challenge during critical periods may result in long-lasting behavioral and neuronal alterations. Nevertheless, our data suggest that immune system activation may expose an underlying genetic vulnerability in *Shank3*^*+/−*^ mice, leading to social behavior deficits.

Exploring the contribution of striatal dysfunctions to ASD pathophysiology allowed us to uncover alterations in specific neuronal populations and to find a novel potential therapeutic target. Specifically, using a circuit-specific knock-down strategy, we identified *Trpv4* upregulation as the link between changes in excitability, inflammatory response, and behavioral deficits. Trpv4 is widely expressed in the brain where it is activated by changes in both osmotic pressure and heat [74–76]. Research into the involvement of Trpv4 in neuropathies and neurodegenerative diseases has attracted an increasing interest [77, 78]. Indeed, whole-genome sequencing of quartet families with ASD has revealed frameshift mutations of *Trpv4* [79], suggesting a possible involvement in the pathogenesis of autism. Furthermore, hyperactivity of these channels occurs in several pathological conditions [49, 78, 80, 81]. Interestingly, Trpv4 activation may induce inflammation by increasing pro-inflammatory cytokines [49, 82] and Trpv4 inhibitors have been used to counteract oedema and inflammation [31]. Although it is well established that inflammatory cytokines may impact both synaptic transmission and neuronal excitability [83, 84], the direct link between Trpv4, neuronal function, and behavior was still relatively unknown. Here, using a circuit approach, we firstly identify an upregulation of *Trpv4* after *Shank3* downregulation. Consequently, based on these results, we found that inflammatory challenge in *Shank3*^*+/−*^ mice increased the expression of *Trpv4* and induced D1R-MSNs hyperexcitability. By rescuing sociability deficits in these mice, we provide a novel link between immunoresponse, genetic background, and neuronal activity in the context of ASD. Finally, our data point at Trpv4 channel as a novel potential candidate for the treatment of ASD symptoms.

Overall, our data highlight that viral-mediated and region-specific ablation of *Shank3*, is a suitable model to obtain mechanistic insights regarding regions and cell types that could be implicated in autism-relevant symptoms and furthermore, to validate hypotheses and potential novel therapeutic interventions.

## Method details

### Mice

The experimental procedures described here were conducted in accordance with the Swiss laws and previously approved by the Geneva Cantonal Veterinary Authority. Male C57BL/6J and D1R:Cre (MMRRC Stock No: 37156-JAX) mice were purchased from The Jackson Laboratory and housed in the institutional animal facility under standard 12 h/12 h light/dark cycles with food and water ad libitum. Drd1a-tdTomato, Shank3Δe4-22 male mice [18] (in this paper Shank3+/+, Shank3+/−, and Shank3−/−) were housed and bred in our animal facility. Experimental animals were group-housed (2–5 per cage) and were behaviorally tested as stated in the corresponding sections in the following paragraphs and on the figure timelines. Younger non-familiar male mice (3–4 weeks; sex-matched) were used as stimuli animals in the three chambers social interaction assay. Behavioral experiments were conducted in a room with fixed low illumination (10–15 Lux) and with controlled humidity (40%) and temperature (22–24 °C). The experiments were always performed within a time frame that started approximately 2 h after the end of the dark circle and ended 2 h before the start of the next dark circle.

### Viruses and stereotactic injections

Viruses used in this study: (1) purified scr*Shank3* and sh*Shank3* (AAV1-GFP-U6-scrmbshRNA; titer: 5.9 × 10^13^ GC/mL and AAV5-ZacF-U6-luczsGreen-sh*Shank3*; titer: 7.4 × 10^13^ GC/mL, Vector Biolabs); (2) AAV5/hsyn-DIO-hM4D(Gi)-mCherry (AV44961, titer: 5.5 × 10^12^ virus molecules/mL, UNC GTC vector core) (3) AAV5-hsyn-eYFP (titer ≥ 7 × 10^12^ virus molecules/mL, Addgene) and AAV5-hSyn1-mTRPV4-2A-eGFP (Vector Biolabs 1.2 × 10^12^ GC/mL); (4) AAV5/2-hSyn1-chl-jGCamp7s-WPRE-SV40p(A) (titer: 7.7 × 10^12^ VG/mL, v406-5, Viral vector ETH Zurich). Viral injections in the NAc were delivered in mice either at an early time-point (at P5 or P6; <P6) or later in life (>P30) depending on the experimental cohort. After anesthesia induction with a mixture of isoflurane/O2, C57Bl/6j wildtype pups or >P30 mice were placed on a stereotaxic frame (Angle One; Leica, Germany). Mice were then locally anesthetized with 50 ul Lidocaine 0.5%, disinfected with Betadine and a small cut is performed. Craniotomy (1 mm in diameter) for >P30 mice is then performed using a surgical micro drill. The virus (150 nL) is injected via a glass micropipette into the region of interest. The skin is closed with sutures and the mouse is let wake up on a heating plate. For the pups, the coordinates used were AP: +3.5 mm, ML: ±0.8 mm, DV: −3.2 mm (measured from lambda), and for >P30 mice, the coordinates were AP: +1.2 mm, ML: ±1.0 mm, DV: −4.4/−4.0 mm (measured from bregma). To obtain bilateral NAc infection, 100 nl of viral solution was infused per injection in pups and 150 nl of viral solution was infused per injection in >P30 mice.

### Social preference and novelty test

Similarly to previous studies [13, 85], a three-chambered social interaction assay was used, comprising a rectangular Plexiglas arena (60 × 40 × 22 cm) (Ugo Basile, Varese, Italy) divided into three chambers (each 20 × 40 × 22 (h) cm). The walls of the center chamber had doors that could be lifted to allow free access to all chambers. The social preference test was performed similarly as published by Moy et al. [86]. Briefly, each mouse was placed in the arena for a habituation period of 10 min, when it was allowed to freely explore the empty arena. At the end of the habituation, the test was performed: two enclosures (16 cm × 9 cm) with metal vertical bars were placed in the center of the two outer chambers. One enclosure was empty (serving as an inanimate object) whereas the other contained a social stimulus (unfamiliar juvenile mouse 25 ± 1 day old). The enclosures allowed visual, auditory, olfactory, and tactile contact between the experimental mice and the mice acting as social stimuli. The juvenile mice in the enclosures were habituated to the apparatus and the enclosures for a brief period of time on the 3 days preceding the experiment. The experimental mouse was allowed to freely explore the apparatus and the enclosures for 10 min. The position of the empty vs. juvenile-containing enclosures alternated and was counterbalanced for each trial to avoid any bias effects.

For some batches of mice (scr- and shShank3), a social novelty test was performed immediately after the social preference test. The empty enclosure was replaced by a new enclosure containing a novel unfamiliar social stimulus (juvenile mouse 25 ± 1 day old). Consequently, the experimental mouse was allowed to freely explore the apparatus and the enclosures for 10 min.

Every session was video-tracked and recorded using Ethovision XT (Noldus, Wageningen, the Netherlands), which provided an automated recording of the time around the enclosures (with virtual zones designed around them), the distance moved, and the velocity. The time spent around each enclosure was assessed and then used to determine the preference score for the social target as compared to the empty enclosure (social/(social + empty)). The arena was cleaned with 1% acetic acid solution and dried between trials. Animals that their total exploration time for both the enclosures was less than 10 s were excluded from the analysis. In particular, one mouse in the D1:Cre^+^::scr*Shank3* group was excluded from the analysis according to this criterion.

In the rescue experiment with the chemogenetic approach, 30 min before the habituation, all scr- and sh*Shank3*-injected mice, regardless of genotype (i.e., D1R:Cre− or D1R:Cre+), were intraperitoneally injected with Clozapine N-oxide (CNO, Cat. No.: BML-NS105-0025, Lot No.: 07131709) dissolved in saline (5 mg/Kg).

In the LPS challenge experiments, *Shank3*^*+/+*^ and *Shank3*^*+/−*^ mice were intraperitoneally injected 24 h before the test with LPS at a dose of 2 mg/Kg in saline (NaCl 0.9%) (Lipopolysaccharides from Escherichia coli O26:B6, Sigma-Aldrich).

### O-maze test

Scr- and shShank3 mice were subjected to an elevated O-maze test for assessing anxiety-like behavior as previously published [87]. Each session lasted 5 min and the surface of the arena was cleaned with 1% acetic acid and dried before testing the next animal. Sessions were video-tracked and recorded using Ethovision XT (Noldus, Wageningen, the Netherlands), which provided an automated recording of the time spent in the open and closed zones of the maze.

### Whole-cell patch clamp recordings

Coronal midbrain slices 250 μm thick containing the NAc were prepared following the experimental injection protocols described above. Brain were sliced in artificial cerebrospinal fluid (aCSF) containing 119 mM NaCl, 2.5 mM KCl, 1.3 mM MgCl2, 2.5 mM CaCl2, 1.0 mM NaH2PO4, 26.2 mM NaHCO3 and 11 mM glucose, bubbled with 95% O2 and 5% CO2. Slices were kept for 20−30 min at 35 °C and then transferred at room temperature. Whole-cell voltage clamp or current clamp electrophysiological recordings were conducted at 32°–34° in aCSF (2–3 ml/min, submerged slices). Recording pipette contained the following internal solution: 140 mM K-Gluconate, 2 mM MgCl2, 5 mM KCl, 0.2 mM EGTA, 10 mM HEPES, 4 mM Na2ATP, 0.3 mM Na3GTP and 10 mM creatine-phosphate. The cells were recorded at the access resistance from 10 to 30 MΩ. MSNs were identified by morphology, the absence of Ih current (500 ms-long voltage-clamp steps down to −140 mV from a holding potential of −70 mV) and a resting membrane potential lower than −60mV. Resting membrane potential (in mV) was read using the Multiclamp 700B Commander (Molecular Devices) while injecting no current (*I* = 0) immediately after breaking into a cell. Action potentials (AP) were elicited in current clamp configuration by injecting depolarizing current steps (50 pA, 500 ms) from 0 to 500 pA, in presence of Picrotoxin (100 µM) and Kynurenic acid (3 mM). For CNO validation and HC-067047 rescue, slices were incubated 20 min with the drugs (CNO 20 µM, HC-067047 10 µM, in DMSO 0.03% final concentration) before starting the excitability protocol. After-hyperpolarization current (AHP) was assessed in voltage clamp configuration by holding the cell at −60mV with a step of +60 mV for 100 ms. TRPV4 currents were assessed by holding the cell at 0 mV followed by a 400 ms ramp from −100 to +100 mV. The ramp protocol was applied every 5 s for 5 min (baseline) and then, the TRPV4 inhibitor, HC067047 (10 µM), was applied and cells were recorded for 20 min. Trpv4 current response was obtained by subtracting the current in the presence of HC067047 from the baseline. For synaptic physiology, MSNs were recorded in aCSF in presence of Picrotoxin (100 µM) and the recording pipette contained the following internal solution: 130 mM CsCl, 4 mM NaCl, 2 mM MgCl2, 1.1 mM EGTA, 5 mM HEPES, 2 mM Na2ATP, 5 mM sodium creatine phosphate, 0.6 mM Na3GTP, 0.1 mM spermine. Excitatory postsynaptic currents (EPSCs) were recorded in voltage-clamp configuration, elicited by placing a bipolar electrode laterally to the NAc. Access resistance (10–30 MΩ) was monitored by a hyperpolarizing step of −4 mV at each sweep, every 10 s. Data were excluded when the resistance changed >20%. The AMPA/NMDA ratio was calculated by dividing the synaptic response at +35 mV, from the response at −70mV. The rectification index (RI) of AMPARs is the ratio of the chord conductance calculated at negative potential (–60 mV) divided by the chord conductance at positive potential (+40 mV) after the AMPA current isolation by D-APV (50 µM) bath application. PPR was measured at −60 mV, with a fixed inter stimulation interval of 50 ms. PPR was calculated by dividing the amplitude of the second EPSC by the amplitude of the first EPSC. The synaptic responses were collected with a Multiclamp 700B-amplifier (Axon Instruments, Foster City, CA), filtered at 2.2 kHz, digitized at 5 Hz, and analyzed online using Igor Pro software (Wavemetrics, Lake Oswego, OR).

### RNA extraction, cDNA synthesis, and RT-PCR

Total RNA was extracted using RNeasy Mini Kit (cat 74104) from QIAGEN. The extraction was performed following the details of the kit. After the extraction, RNA quantification was performed using NanoDrop 1000 (Thermo Scientific) and the samples were stored at −80 °C until cDNA synthesis. RNA integrity was checked using the Agilent 2100 Bioanalyzer (RIN was always > 8). cDNA synthesis for two-step RT-PCR was performed using the QuantiTect Reverse Transcription Kit (cat 205313) from QIAGEN. For each sample, 1 ug of RNA was retrotranscribed in cDNA following the kit instruction. 200 ng of cDNA was used for the RT-PCR analysis using a Sybr Green technology. Plates were processed on the 7900HT systems from Thermo Fisher Scientific, equipped with automated devices for plates loading. (Tecan Freedom EVO). SHANK3 forward primer 5′ gtagccacctcttgctcacat 3′, reverse primer 5′ ttgccaaccattctcatcagt 3′; IL-1β forward primer 5′ caaccaacaagtgatattctccatg 3′, reverse primer 5′ gatccacactctccagctgca 3′; TNF-α forward primer 5′ gacgtggaactggcagaagag 3′, reverse primer 5′ gccacaagcaggaatgagaag 3′; Trpv4 forward primer 5′ gtctcgcaagttcaaggact 3′, reverse primer 5′ aaacttacgccacttgtctc 3′; HCN1 forward primer 5′ gaaatggttaatgattcctggg 3′, revers primer 5′ cgaaagggagtaaagacgac 3′; Trpv1 forward primer 5′ aaggctctatgatcgcagga 3′, reverse primer 5′ cagattgagcatggctttga 3′; Actin forward primer 5′ agagggaaatcgtgcgtgac 3′, reverse primer 5′ caatagtgatgacctggccgt 3′. Reactions were carried out using iTaq™ Universal SYBR^®^ Green Supermix (Biorad) by 50 °C for 2 min, 95 °C for 10 min followed by 40 cycles at 95 °C for 15 s and 60 °C for 1 min. Relative quantification of gene expression was performed according to the ΔΔ−Ct method [88].

### FACS sorting and RNA sequencing

Mice were anesthetized in isoflurane and decapitated to dissect fresh brains in ice-cold aCSF (see above). Brains were kept in ice-cold and O_2_ 95%, CO_2_ 5% bubbled aCSF during the preparation of coronal slices, 300 μm thick using a vibratome. Selected slices were used to manually microdissect the NAc using a total of 4–5 P30 mice for each experiment. The dissected tissue was moved in 1.5 ml FACS buffer (L15 added with Glucose 2 mg/ml, Bovine Serum Albumin 0,1%, Citrate Phosphate Dextrose 16.7%, DNAseI 10 U/ml). After removing the FACS buffer, the tissue was incubated in 400 μl of L15 0.01%Papain (Worthington, #LS003118) and incubated 30′ at +37 °C. The tissue was mechanically disrupted pipetting 10 times with a P1000 and a P200 sterile tip, and Papain digestion was blocked by adding FACS buffer 0.02% Chicken egg white inhibitor (Sigma, #T9253). The cell suspension was passed through a 70 µm strainer (ClearLine, # 141379C) and spun at 200 g for 5′ at +4 °C. The precipitate was resuspended in 1 ml FACS buffer, this step was repeated a second time to further wash cellular debris. 8 μl of Hoechst (0.1 mg/mL) were added to the sample and incubated for 7′ at +37 °C. Before FACsorting we added the 5 μl of the cellular death dye Draq7TM (Viability dye, Far-red DNA intercalating agent, Beckman Coulter, #B25595). The suspension was sorted on an Astrios II cell sorter (Beckam Coulter), enriching for Hoechst stained and Draq7TM non-stained particles. Forward and side scatter were used to exclude smaller cellular debris and duplets. 488 and 568 nm laser excitation were used to separate the desired combinations of cellular population. Each cell population was sorted in FACS buffer and spun down at 200 g for 5′ to be dried and snap-frozen in liquid nitrogen before RNA extraction. FACsorting experiments were performed within the Flow cytometry facility at the University of Geneva.

### Sequencing libraries preparation

To prepare cDNA libraries collected frozen tissue was processed using a QIAGEN RNeasy kit (QIAGEN, #74034) to extract RNA and prepare cDNA libraries using SMARTseq v4 kit (Clontech, # 634888) and sequenced using HiSeq 2500 in 100 pairbase length fragments for a minimum of 1 million reads per sample. Sequences were aligned using STAR aligner [89] using the mouse genome reference (GRCm38). The number of read per transcripts was calculated with the open-source HTseq Python library [90]. All analyses were computed on the Vital-it cluster administered by the Swiss Institute of Bioinformatics. Sequencing experiments were performed within the Genomics Core Facility of the University of Geneva.

### Sequencing analysis

Count tables were normalized to reads per million (RPM) and genes were filtered keeping only those with more than 10 RPM (Supplementary Information Table S1). DEseq2 package was used to normalize samples to RPM count tables. In Fig. 3b differentially expressed genes were selected on a worst-case scenario threshold of 1.5 fold, keeping the data from the replicates corresponding to the pair that gave the minimum fold change between each pair of conditions tested. The full list of results for the worst-case scenario fold change analysis is shown in Supplementary Fig. 5a. We performed PCA analysis with all of the samples and all of the genes selected above the worst-case scenario threshold of 1.5 (855 genes, supplementary information Table S2); these data were normalized by rlog transformation from the DEseq2 package and then used for PCA analysis. SFARI genes (https://gene.sfari.org/tools) belonging to the list of significantly altered genes in AAV-scr*Shank3* versus AAV-sh*Shank3* infected D1R-tom+ and D1R^−^-tom-samples are plotted in Supplementary Fig. 5b, c and have been tested for enrichment using Fisher test in Fig. 3d, the 178 worst case scenario differentially expressed genes in AAV-sh*Shank3* versus AAV-scr*Shank3* D1R-tom+ samples, split in sh upregulated and sh downregulated were analyzed for significantly enriched GO:Terms using GOrilla [91] and the REViGO [92] online tools, selecting GO:Terms with adjusted *P*-value lower than 1^e−3^.

Gene expression heatmap in Fig. 3e was produced normalizing the rlog transformation of RPM count tables and allowing samples and genes to cluster by Euclidean distance (Supplementary Information Table S3). All analyses have been made using R, packages used: DEseq2 [93], reshape2 [94], ggplot2 [95], scater [96], IHW [97]. Count table and FASTQ files are available at the GEO database (GSE139683).

### Optic fiber implantation and fiber photometry system

As explained in the subchapter “Viruses and stereotactic injections”, adult mice previously injected with AAV5/2-hSyn1-chl-jGCamp7s-WPRE-SV40p(A) in the NAc were placed on a stereotaxic frame (Angle One; Leica, Germany). Unilateral craniotomy (ø 1 mm) was then performed with the following stereotactic coordinates: AP: +1.2 mm, ML: ±1 mm (measured from bregma). An optic fiber (ø 200 μm) was then implanted into the NAc (DV: −4.2 mm) and fixed on the skull with dental acrylic. All animals underwent behavioral experiments 3−5 weeks after surgery. For recording the calcium transients during the three-chamber test (10 min of habituation and 10 min of social preference test, the mice were connected to the fiber photometry system through a multimode patch cable (FC-MF1.25, ø 200 μm, Doric lenses inc.) which could be reversibly attached and detached to the implanted optic fiber in the mouse brain.

The fiber photometry system (Doric lenses inc.) consisted of two excitation channels. A 465 nm LED (CLED_465, Doric lenses inc.) was used to extract a Ca^2+^-dependent signal, and a 405 nm LED (CLED_405, Doric lenses inc.) was used to obtain a Ca^2+^-independent isosbestic signal. Light from the LEDs was directed through a fluorescence MiniCube composed of 4 ports with 1 integrated photodetector head (iFMC4_AE(40 5)_E(460-490)_F(500-550) _S, Doric lenses inc.). Light emissions from GCamp7s expressing neurons were then collected back through the optic fiber, and directed through a detection path, passing a dichroic mirror to reach the photodetector integrated in the MiniCube. A fiber photometry console (FPC, Doric lenses inc.) and the Doric software (version 5.4.1.5) were used to control the LEDs and acquire fluorescence data at 12 kHz. LEDs were alternately turned on and off at 40 Hz in a square pulse pattern.

### Analyses of fiber photometry data

Fiber photometry data were analyzed using custom R codes which are available upon request. For each experiment, we defined the *F*_0_ as the baseline activity recorded during the habituation, 5 min before the presentation of the stimuli. The fluorescence change was determined as Δ*F*/*F* and calculated as (*F* − *F*_0_)/*F*_0_ where *F* is the fluorescence at each bin. The acquisition frequency was at 12 kHz (bins of 1/12158 s) for the entire recordings. The construction of Peri-event time histogram (PETH) was made by aligning and centering fiber photometry data on specific events. These events were obtained by the automated tracking of the mouse body parts performed by Ethovision XT (Noldus, Wageningen, the Netherlands). The events detected were: the entry in proximity of the empty or juvenile enclosures (see Fig. 5b-b**′**). The virtual circular zone that defines the proximity to an enclosure had the following diameter: ø = ø_encolure_ + 5 cm and were centered on the enclosures. Events that happened in the first and last 10 min of the test were not taken into account in the analysis. We used Z-score to normalize Δ*F*/*F* computed as (mean_(Δ*F*/*F*)_ − *µ*_(Δ*F*/*F*)_)/σ_(Δ*F*/*F*)_, where mean_(Δ*F*/*F*)_ is the averaged Δ*F*/*F* at each bin of the PETH, *µ*_(Δ*F*/*F*)_ the averaged Δ*F*/*F* of the whole session and σ_(Δ*F*/*F*)_ the Δ*F*/*F* standard deviation of the whole session.

### Cannulations and intra-NAc microinfusions

As explained in the subchapter “Viruses and stereotactic injections” adult mice (P50-60) were placed on a stereotaxic frame (Angle One; Leica, Germany). Bilateral craniotomy (ø1 mm) was then performed with the following stereotactic coordinates: AP: +1.2 mm, ML: ±1 mm, DV: −3.8 mm (measured from bregma). Bilateral stainless steel 26-gauge cannula (5 mm ped, PlasticsOne, Virginia, USA) was implanted above the NAcs and fixed on the skull with dental acrylic. Between experiments, the cannula was protected by a removable cap in aluminum. All animals underwent behavioral experiments 1–2 weeks after surgery. In the rescue experiment with the Trpv4 antagonist, cannulated scr- or sh *Shank3* and *Shank3*^*+/−*^ mice were infused 10 min before the three-chamber task (Fig. 3h, more precisely, 10 min before the habituation in the arena). Cannulated scr- and sh*Shank3* mice performed the behavioral task two times with 7-days pause period between the trails. sh*Shank3* injected mice were randomly infused with 2 µL (500nL m^−1^) of vehicle (~3 % dimethyl sulfoxide (DMSO, Sigma) diluted in aCSF) or with 2 µL Trpv4 antagonist (HC-067047, Sigma 2 µg diluted in aCSF-DMSO ~3 %). The treatment was counterbalanced between trials. On the other hand, scr*Shank3* injected mice were infused both trials with vehicle. Similarly, cannulated *Shank3*^*+/−*^ mice were intraperitoneally injected with LPS at a dose of 2 mg/Kg 24 h before the test. Then mice were randomly infused 10 min before the test with 2 µL (500 nL m^−^^1^) of vehicle (~3 % dimethyl sulfoxide (DMSO, Sigma) diluted in aCSF) or with 2 µL Trpv4 antagonist (HC-067047, Sigma 2 µg diluted in aCSF-DMSO ~3%).

### Tissue processing for post hoc studies

For post hoc analysis, adult mice were anesthetized with pentobarbital (Streuli Pharma) and sacrificed by intracardial perfusion of 0.9% saline followed by 4% PFA (Biochemica). Brains were post-fixed overnight in 4% PFA at 4 °C. 24 h later, they were washed with PBS before 50 μm thick vibratome cutting. After each behavioral experiment, post hoc analysis was performed to validate the localization of the infection and/or cannulation.

### Immunohistochemistry and image acquisition

Prepared slices were washed three times with phosphate buffered saline (PBS) 0.1 M. Slices were then pre-incubated with PBS-BSA-TX buffer (0.5% or 3% BSA and 0.3% Triton X-100) for 90 min at room temperature in the dark. Subsequently, cells were incubated with primary antibodies diluted in PBS-BSA-TX (0.5% BSA and 0.3% Triton X-100) overnight at 4 °C in the dark. The following day slices were washed three times with PBS 0.1 M and incubated for 90 min at room temperature in the dark with the secondary antibodies diluted in PBS-BSA buffer (0.5% BSA). Finally, coverslips were mounted using fluoroshield mounting medium with DAPI (Abcam, ab104139).

Primary antibody used in this study: polyclonal rabbit anti-Kir3.1 (Girk1, 1/750 dilution, Alamone labs, APC-005), and polyclonal rabbit anti-cFos (1/500 dilution, Oncogene, 226003). Secondary antibody used at 1/500 dilution: donkey anti-rabbit 488 (Alexa Fluor, Abcam ab150073). Post hoc tissue images were acquired using a confocal laser-scanning microscope LSM700 (Zeiss) or an Axiocam fluo wide field microscope (Zeiss) depending on the size of the ROI.

### SDS-PAGE and Western Blot analyses

NAc and Striatum were dissected from mice infected with shShank3 or scrShank3 virus. Samples were homogenized at 4 °C with ice-cold lysis buffer (Tris-HCl 50 mM, NaCl 150 mM, Triton 1%) with protease inhibitors (Roche cOmplete™ Protease Inhibitor Cocktail), phosphatase inhibitors (PhosSTOPTM, Roche Diagnostics GmbH) using sequential needle syringe 20G, 26G, and 30G. Protein concentration was calculated using BCA protein assay kit (Thermo Scientific) and samples were separated by electrophoresis onto NuPAGE Bis-Tris 4−12% gel (#NP0322PK2, Invitrogen) and transferred to a nitrocellulose membrane. After 45 min of blocking with TBST-BSA 5%, membranes were probed overnight with primary antibody (anti-Shank3 Neuromab clone N367/62 1:1000, anti-GFP Millipore #MAB3580 1:5000, anti-Tubulin Sigma-Aldrich #T5168 1:10000) diluted in TBST-BSA 1%, followed by incubation with appropriate horseradish peroxidase-conjugated secondary Ab (#1706516 dilution 1:10000 in TBST-BSA 1%). Blots were developed using WesternBright ECL (Advansta) and the chemiluminescence signal was visualized using Fusion solo S system (VILBER). Blots were quantified using ImageJ software.

### Statistical analysis

Statistical analysis was conducted with GraphPad Prism 7 and 8 (San Diego, CA, USA) and SPSS version 21.0 (IBM Corp, 2012). Statistical outliers were identified with the ROUT method (*Q* = 1) and excluded from the analysis. The normality of sample distributions was assessed with the Shapiro–Wilk criterion and when violated non-parametric tests were used. When normally distributed, the data were analyzed with independent t-tests, one sample t-tests, one-way ANOVA, and repeated measures (RM) ANOVA as appropriate. When normality was violated, the data were analyzed with Mann–Whitney or Wilcoxon tests, while for multiple comparisons, Kruskal–Wallis or Friedman test was followed by Dunn’s test. For the analysis of variance with two factors (two-way ANOVA, RM two-way ANOVA, and RM two-way ANOVA by both factors), normality of sample distribution was assumed, and followed by Sidak or Tukey post hoc test. Data are represented as the mean ± SEM and the significance was set at 95% of confidence.

## Supplementary information

supplementary figures

Supplelemtary figure and table legends

Supplementary table 1

Supplementary table 2

Supplementary table 3

## Data Availability

All the data are in the manuscript or in supplementary material. Videos, behavioural scoring, and code analysis will be made available upon request.

## Code availability

All the codes use in this study are available upon request.
