# Supplementary material for: Inhibition of Trpv4 rescues circuit and social deficits unmasked by acute inflammatory response in a Shank3 mouse model of Autism
Source: Mol Psychiatry. 2022 Jan 12;27(4):2080–94. doi: 10.1038/s41380-021-01427-0 (PMC9126815; doi:10.1038/s41380-021-01427-0)
Supplement: Supplementary file 1 — supplementary figures [file 41380_2021_1427_MOESM1_ESM.pdf]

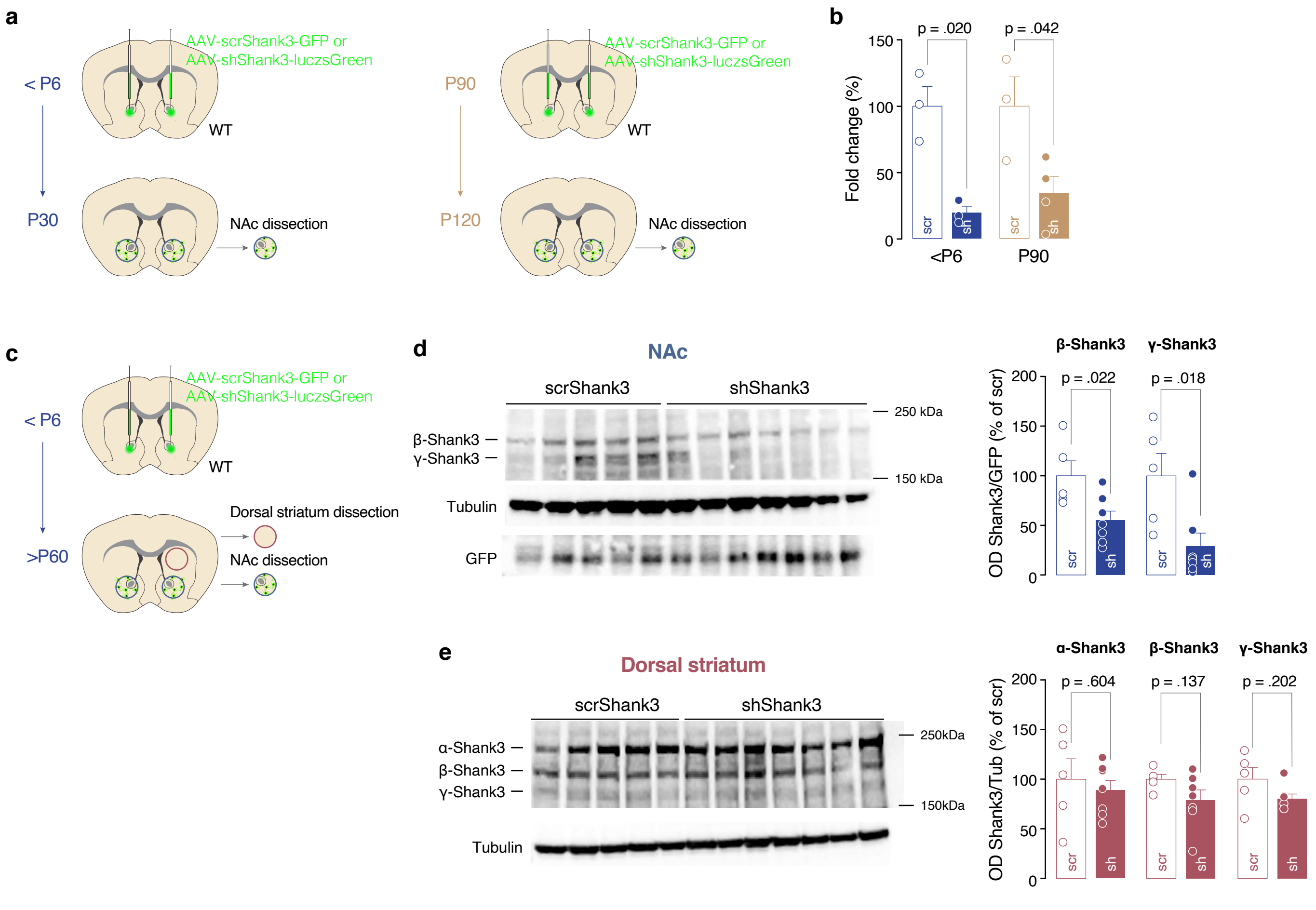

Sup. Figure 1 - Tzanoulinou, Musardo, Contestabile et all

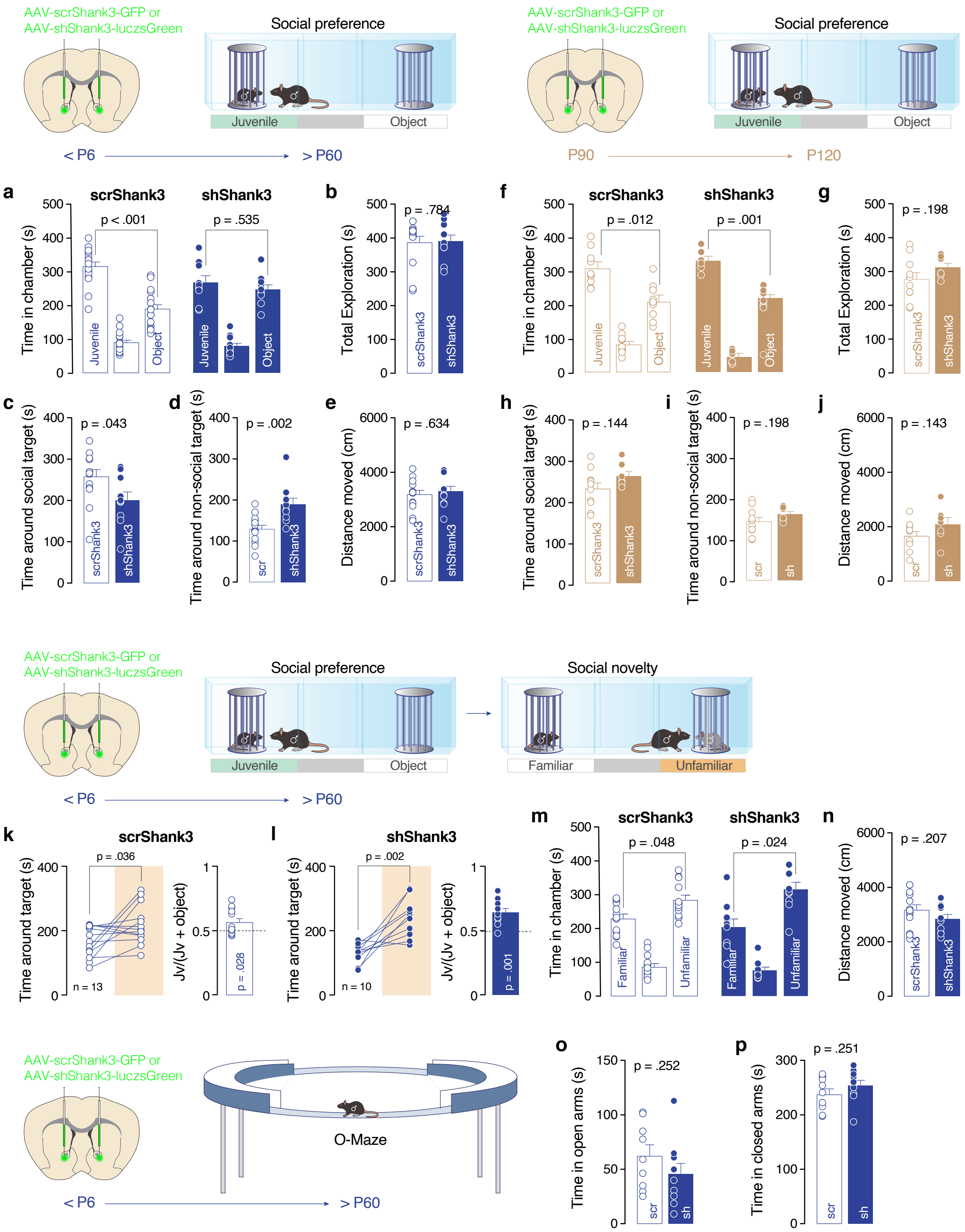

Sup. Figure 2 - Tzanoulinou, Musardo, Contestabile et all

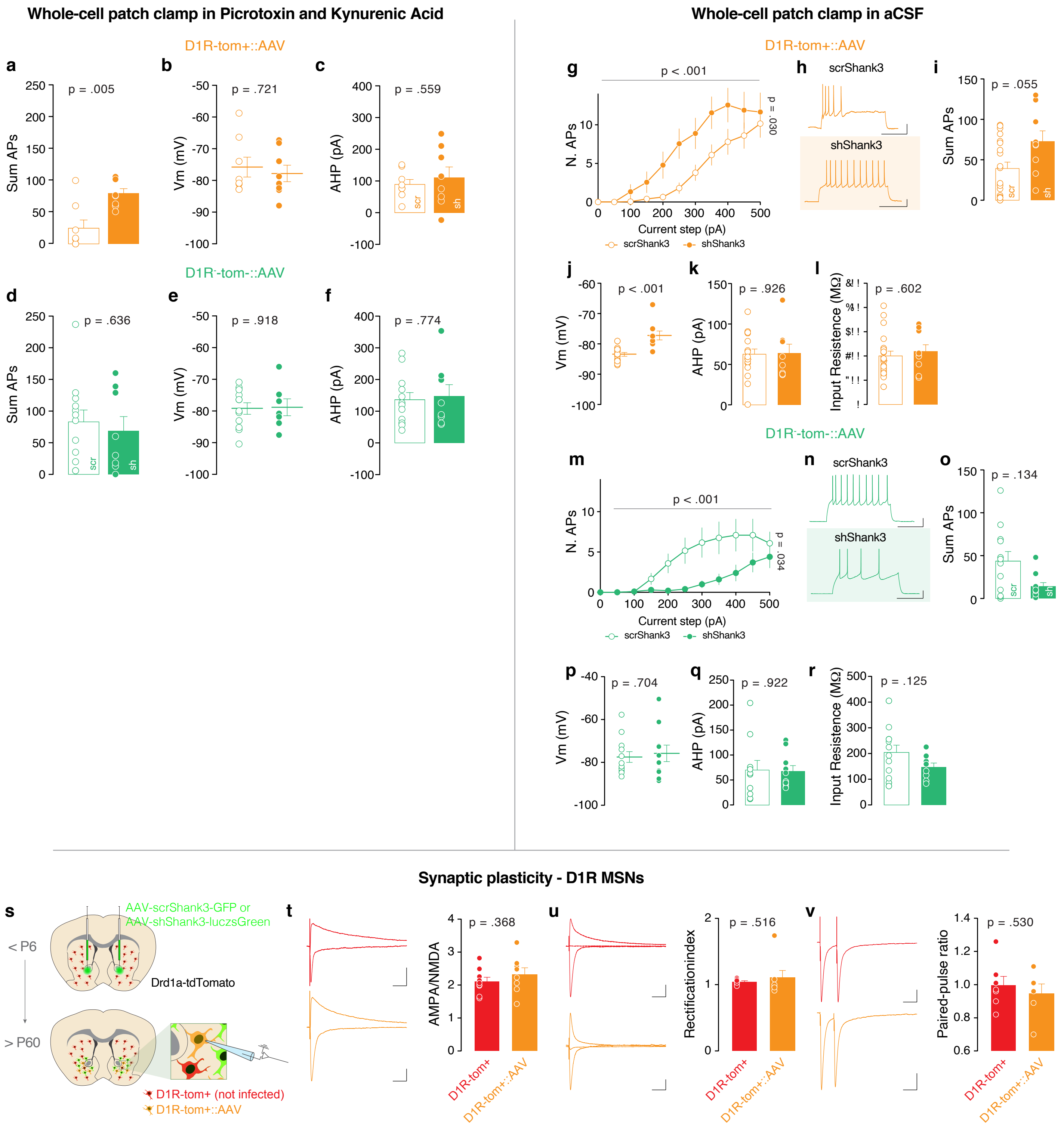

Sup. Figure 3 - Tzanoulinou, Musardo, Contestabile et all

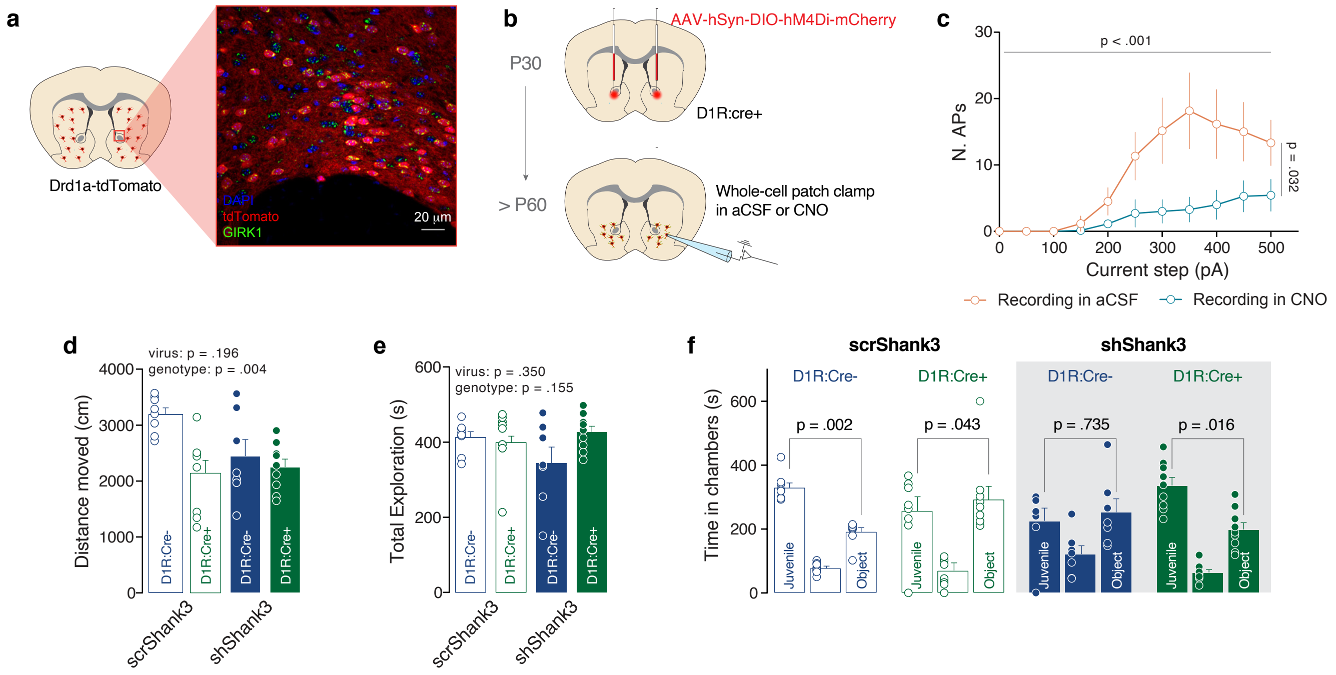

Sup. Figure 4 - Tzanoulinou, Musardo, Contestabile et all

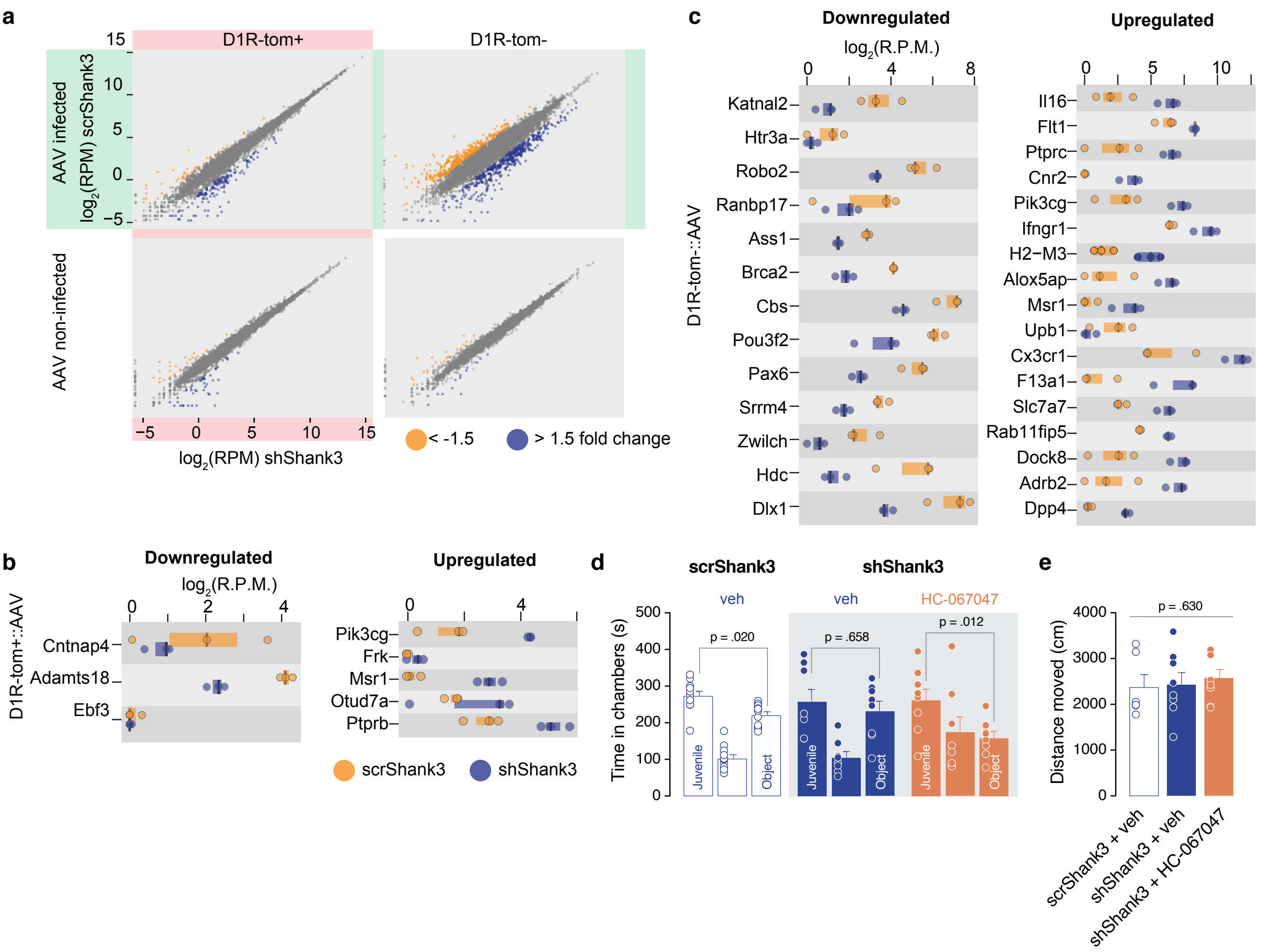

Sup. Figure 5 - Tzanoulinou, Musardo, Contestabile et all

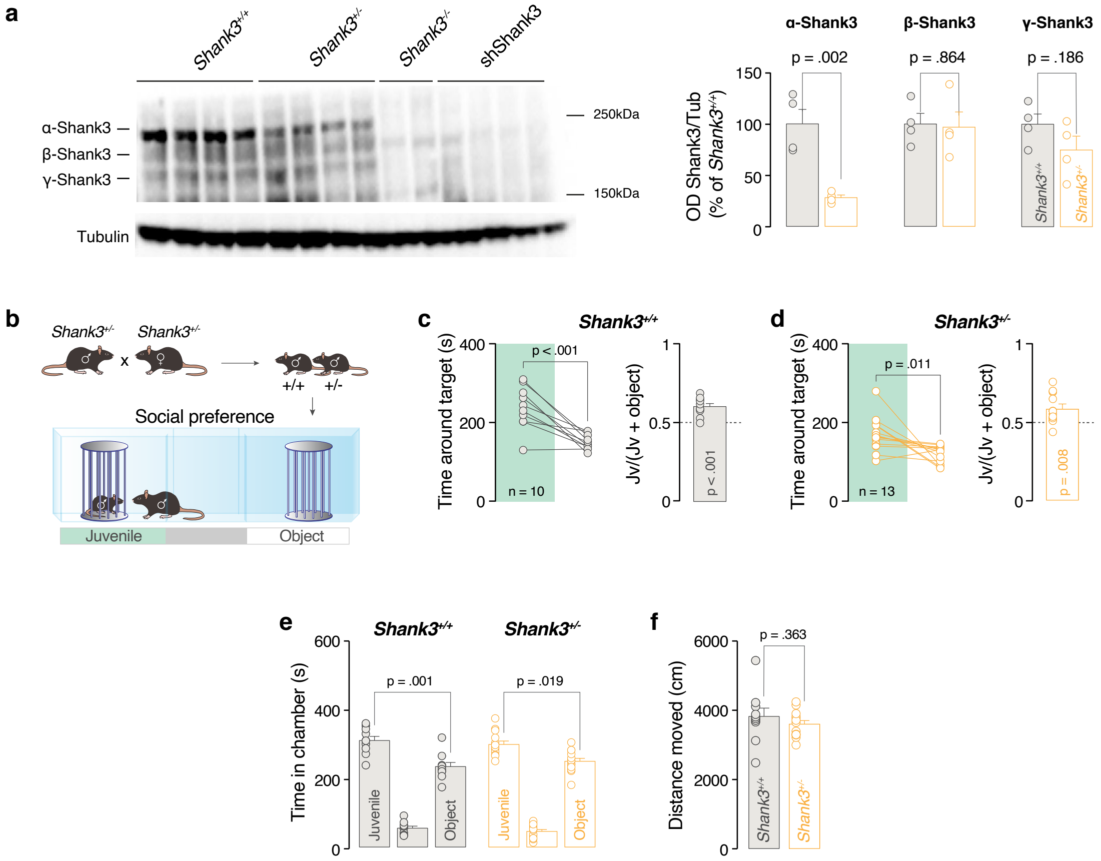

Sup. Figure 6 - Tzanoulinou, Musardo, Contestabile et all

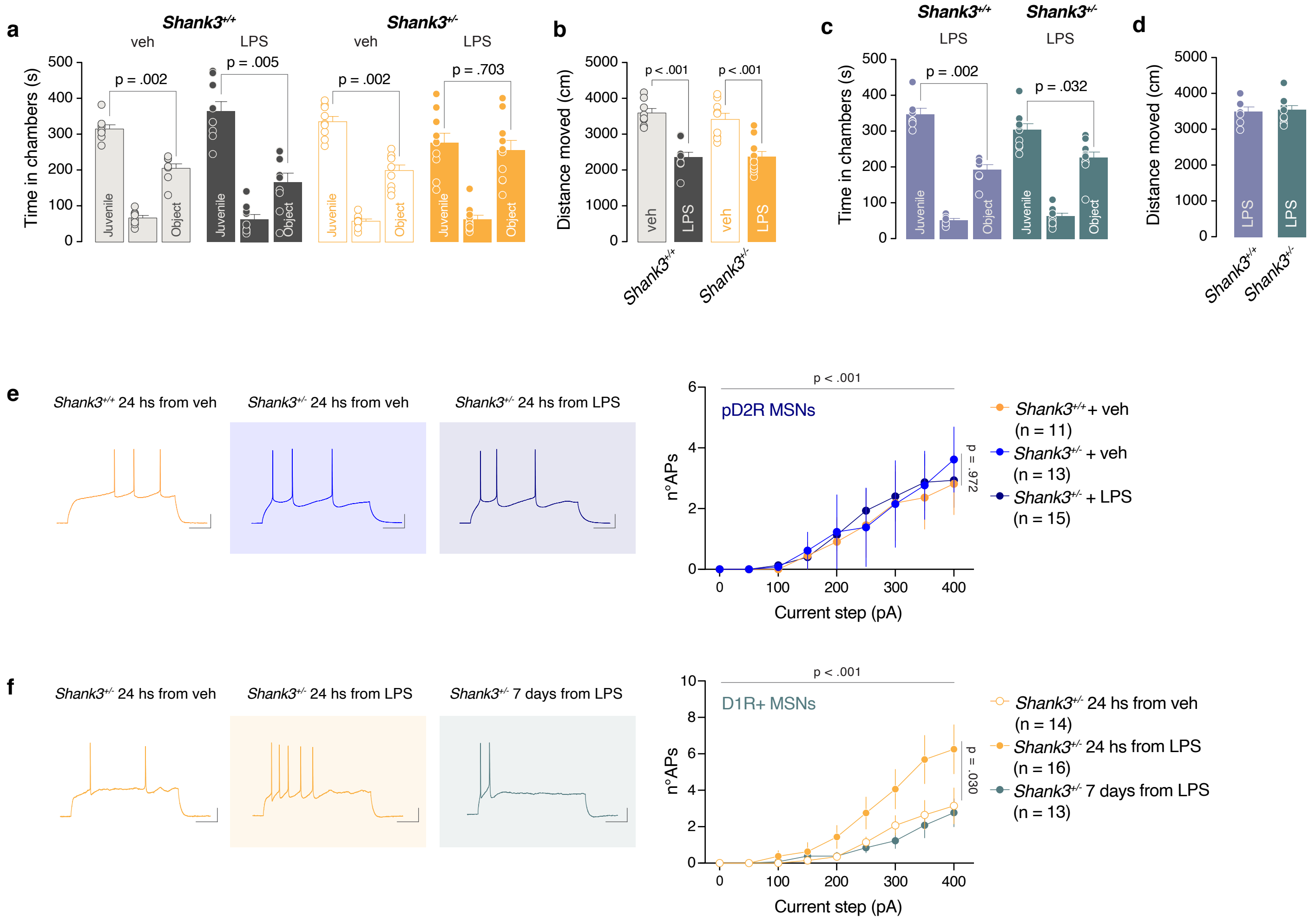

Sup. Figure 7 - Tzanoulinou, Musardo, Contestabile et all

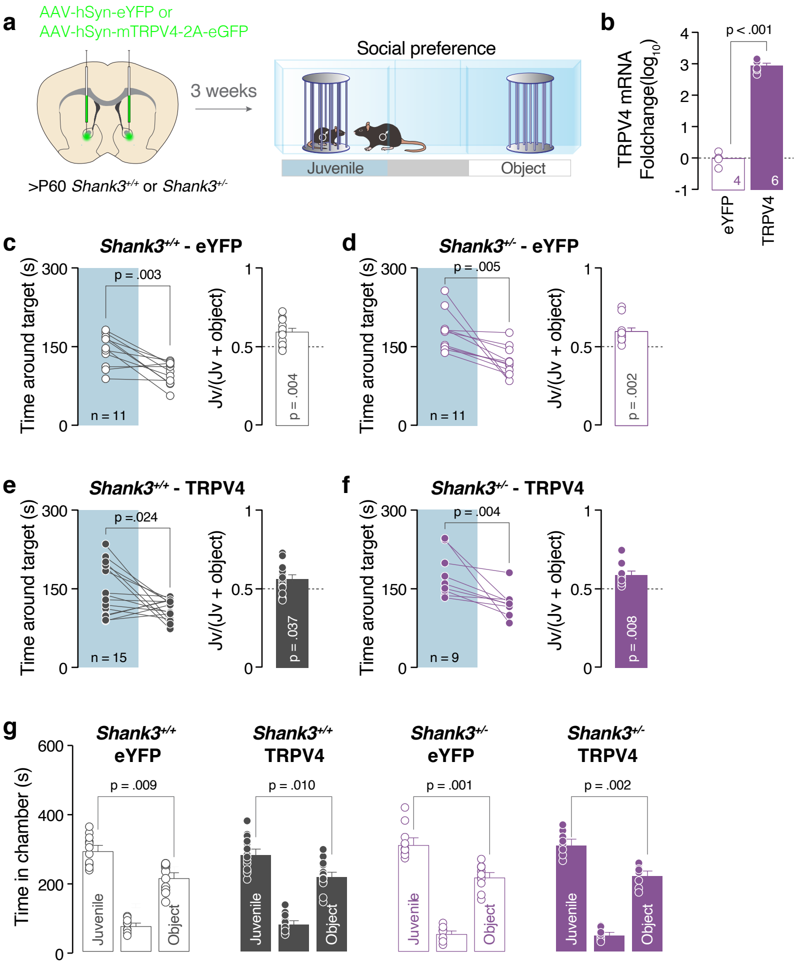

Sup. Figure 8 - Tzanoulinou, Musardo, Contestabile et all

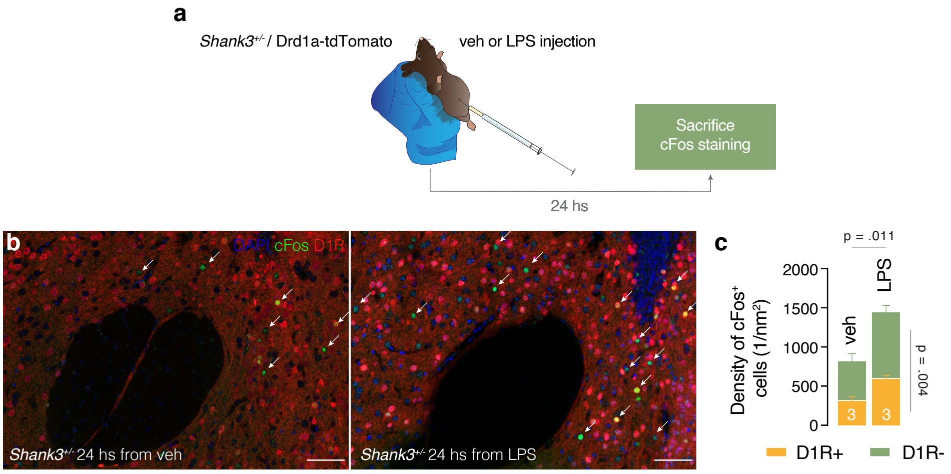

Sup. Figure 9 - Tzanoulinou, Musardo, Contestabile et all

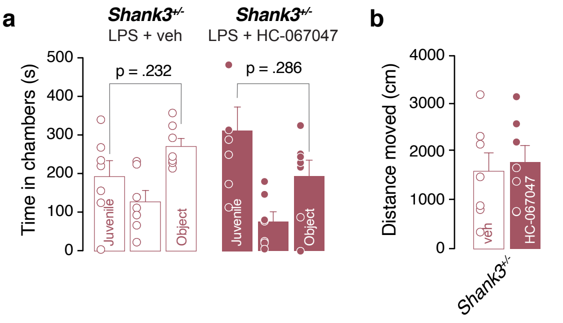

Sup. Figure 10 - Tzanoulinou, Musardo, Contestabile et all
