# Supplementary material for: Inhibition of Trpv4 rescues circuit and social deficits unmasked by acute inflammatory response in a Shank3 mouse model of Autism
Source: Mol Psychiatry. 2022 Jan 12;27(4):2080–94. doi: 10.1038/s41380-021-01427-0 (PMC9126815; doi:10.1038/s41380-021-01427-0)
Supplement: Supplementary file 2 — Supplelemtary figure and table legends [file 41380_2021_1427_MOESM2_ESM.docx]

**Supplementary Figure 1: Validation of AAV-sh*Shank3-*luczsGreen virus**

**(a)** Schema of injection sites in the NAc with AAV-scrShank3-GFP or AAV-shShank3-luczsGreen in P6 (left) or P90 (right) mice. Subsequently, the NAc was dissected and mRNA was extracted. **(b)** Real-time PCR analysis of NAc dissected from P6- or P90-injected mice confirm the downregulation of *Shank3* in sh-infected mice (two-way ANOVA followed by Bonferroni’s multiple comparisons test: virus main effect *F_(1, 8)_* = 18.80, *p* = 0.003*).* **(c)** Schema of injection sites in the NAc with AAV-scrShank3-GFP or AAV-shShank3-luczsGreen in P6. Subsequently, the NAc and the dorsal striatum were dissected during adulthood and WB was performed. **(d)** Left: representative western-blot images from NAc homogenate. Right: Optical density (OD) quantification (unpaired-samples t-test: β-Shank3 *t*_(10)_ = 2.695, *p* = 0.022. Mann-Whitney test: γ-Shank3 *U* = 3, *p* = 0.018). **(e)** Left: representative western-blot images from dorsal striatum homogenate. Right: Optical density (OD) quantification (unpaired-samples t-tests: α-Shank3 *t*_(10)_ = 0.536, *p* = 0.604; β-Shank3 *t*_(10)_ = 1.616, *p* = 0.137. Mann-Whitney test: γ-Shank3 *U* = 9, *p* = 0.202). Error bars report SEM.

**Supplementary Figure 2: *Shank3* downregulation during development increases interaction with the non-social target and does not affect social novelty and anxiety.**

**(a** and **f)** Time spent in compartments for mice injected ≤ P6 and at adulthood (paired-samples t-tests for object- vs. social-containing chambers: (a) P6 mice: scrShank3 *t* _(12)_ = 5.047, *p* < 0.001), shShank3 *t*_(9)_ = 0.645, *p* = 0.535; (f) P90 mice: scrShank3 *t*_(9)_ = 3.144, *p* = 0.012, shShank3 *t*_(6)_ = 5.686, *p* = 0.001). **(b** - e) other measurements in the 3-chamber for neonatally-injected mice. (b) Total exploration time around the enclosures (Mann-Whitney test, *p* = 0.784). **(c)** Time spent around the enclosure containing the social stimulus (unpaired t-test: *t*_(21)_ = 2.152, *p* = 0.043). **(d)** Time spent around the non-social target (unpaired t-test: *t*_(21)_ = 3.499, *p* = 0.002). **(e)** Distance moved in the apparatus (unpaired t-test: *t*_(21)_ = 0.483, *p* = 0.634). **(g - j)** other measurements in the 3-chamber for adulthood- injected mice. (g) Total exploration time around the enclosures (unpaired t-test: *t*_(15)_ = 1.347, *p* = 0.198). **(h)** Time spent around the enclosure containing the social stimulus (unpaired t-test: *t*_(15)_ = 1.541, *p* = 0.144). **(i)** Time spent around the non-social target (unpaired t-test: *t*_(15)_ = 1.347, *p* = 0.198). **(j)** Distance moved in the apparatus (unpaired t-test: *t*_(15)_ = 1.544, *p* = 0.143). **(k** and **l)** Left: time spent around the enclosures during the social memory test for mice injected at ≤P6 (Paired-samples t-tests for unfamiliar- vs. familiar mouse: (k) *t*_(12)_ = 2.356, *p* = 0.036; (l) *t*_(9)_ = 4.342, *p* = 0.002). Right: unfamiliar preference index for mice injected at ≤ P6 (one-sample t-tests against chance level = 0.5: (k) scr*Shank3* *t*_(12)_ = 2.461, *p* = 0.028; (l) sh*Shank3* *t*_(9)_ = 4.627, *p* = 0.001). **(m)** Time spent in compartments during the social memory test for mice injected ≤ P6 (paired-samples t-tests for object- vs. social-containing chambers: scrShank3 *t*_(12)_ = 2.197, *p* = 0.048; shShank3 *t*_(9)_ = 2.715, *p* = 0.024). **(n)** Distance moved in the apparatus during social memory test (unpaired t-test: *t*_(21)_ = 1.302, *p* = 0.207). **(o)** Time spent in the open arms during the O-maze test (unpaired t-test: *t*_(17)_ = 1.185, *p* = 0.252). **(p)** Time spent in the closed arms during the O-maze test (unpaired t-test: *t*_(17)_ = 1.189, *p* = 0.251). Error bars report SEM.

**Supplementary Figure 3: *Shank3* NAc downregulation alters D1R MSNs excitability without affecting synaptic plasticity.**

**(a, d, i** and **o)** Total number of APs across all steps ((a) Mann Whitney test, *p =* 0.005. (d) unpaired t-test, *t_(18)_ =* 0.482 *p =* 0.636. (i) Mann Whitney test, *p =* 0.055. (o) Mann Whitney test, *p =* 0.134). **(b, e, j** and **p)** Resting membrane potential of recorded cells ((b) Mann Whitney test, *p =* 0.721. (e) unpaired t-test, *t_(17)_ =* 0.105, *p =* 0.918. (j) Mann Whitney test, *p <* 0.001. (p) unpaired t-test, *t*_(20)_ = 0.385, *p =* 0.704). **(c, f, k** and **q)** After-hyperpolarization current (AHP) of recorded cells ((c) unpaired t-test, *t_(14)_ =* 0.597, *p =* 0.559. (f) unpaired t-test, *t_(18)_ =* 0.291, *p =* 0.774. (k) unpaired t-test, *t_(24)_* = 0.094*,* *p =* 0.926. (q) unpaired t-test, *t*_(18)_ = 0.099, *p =* 0.922). **(g)** Number of action potentials (nAPs) across increasing depolarizing current steps (0-500 pA) for D1R-tom+::scrShank3 and shShank3 MSNs (repeated measures (RM) two-way ANOVA, main effect of virus *F_(1, 27)_* = 5.285, *p =* 0.030, main effect of current steps *F_(10, 270)_ =* 32.46, *p <* 0.001, virus by current steps interaction *F_(10 , 270)_ =* 1.957, *p =* 0.038, n = 9 cells, 3 mice (shShank3), n = 20 cells, 5 mice (scrShank3)). **(h)** Example traces from 300 pA depolarizing current injection in D1R-tom+ MSNs infected with scrShank3 (upper part) or with shShank3 (lower part). **(l, r)** Input resistance of recorded cells (unpaired t-test: (l) *t_(27)_ =* 0.528, *p =* 0.602; (r) *t*_(19)_ = 1.607, *p* = 0.125). **(m)** Number of action potentials (nAPs) across increasing depolarizing current steps (0-500 pA) for D1R-tom-::scrShank3 and shShank3 MSNs (repeated measures ANOVA, main effect of virus *F_(1, 20)_ =* 5.207, *p =* 0.034, main effect of current steps *F_(10, 200)_ =* 11.77, *p <* 0.001, virus by current steps interaction *F_(10, 200)_ =* 2.958 *p* = 0.002, n = 10 cells, 3 mice (shShank3), n = 12 cells, 3 mice (scrShank3)). **(n)** Example traces from 300 pA depolarizing current injection in D1R-tom- MSNs infected with scrShank3 (upper part) or with shShank3 (lower part). **(s)** Experimental design. Drd1a-dTomato mice were injected neonatally in the NAc with scr or sh*Shank3* virus and whole-cell patch clamp recordings were performed during early adulthood. **(t)** AMPA/NMDA ratio (-70/+35 mV, unpaired t-test: *t_(15)_ =* 0.927, *p =* 0.368). **(u)** Rectification index (Mann Whitney test: *p =* 0.516). **(v)** Paired-pulse ratio (Unpaired t-test: *t_(11)_ =* 0.649, *p =* 0.530). Error bars report SEM.

**Supplementary Figure 4: Dampening D1R-MSNs activity improves social deficits in NAc-shShank3 mice.**

**(a)** Representative image of GIRK1 expression (green) in the NAc of Drd1a-dTomato (red) mice. **(b)** Experimental design. D1R-Cre positive (D1R:Cre^+^) mice were injected in the NAc with AAV-hSyn-DIO-hM4Di-mCherry (DREADD) and after P60 whole-cell patch clamp recordings were performed. NAc slices were either pre-incubated with CNO and recorded in presence of CNO, or were incubated and recorded in aCSF only. **(c)** Number of action potentials (nAPs) across increasing depolarizing current steps (0-500 pA) in presence or absence of CNO. The number of APs was significantly decreased by the bath application of CNO (repeated measures ANOVA, main effect of drug *F_(1, 11)_ =* 6.060, *p* = 0.032, main effect of current steps *F_(10, 110)_ =* 12.11, *p <* 0.001, drug by current steps interaction *F_(10, 110)_* = 4.342, *p* < 0.001, n = 6 cells (aCSF), 7 cells (CNO); n = 2 mice). **(d)** Distance moved for D1R:Cre+/- mice injected with DREADD and scrShank3 or DREADD and shShank3 (two-way ANOVA: main effect of virus *F*_(1, 28)_ = 1.756, *p* = 0.196, main effect of genotype *F*_(1, 28)_ = 10.039, *p* = 0.004, virus by genotype interaction *F*_(1, 28)_ = 4.959, *p* = 0.034). **(e)** Total exploration time for D1R:Cre^+^ or D1R:Cre^-^ mice injected with DREADD and scrShank3 or DREADD and shShank3 (two-way ANOVA: main effect of virus *F*_(1, 28)_ = 0.902, *p* = 0.350, main effect of genotype *F*_(1, 28)_ = 2.135, *p* = 0.155, virus by genotype interaction *F*_(1, 28)_ = 2.604, *p* = 0.118). **(f)** Time spent in compartments of the three-chamber social interaction task for D1R:Cre^+^ or D1R:Cre^-^ mice injected with DREADD and scrShank3 or DREADD and shShank3 (paired-samples t-tests for object- vs. social-containing chambers: D1R:Cre^-^::scrShank3: *t*_(7)_ = 4.916, *p* = 0.002; D1R:Cre^+^::scrShank3: *t*_(7)_ = 0.043, *p* = 0.967; D1R:Cre^-^::shShank3: *t*_(6)_ = 0.355, *p* = 0.735; D1R:Cre^+^::shShank3: *t*_(8)_ = 3.031, *p* = 0.016). Error bars report SEM.

**Supplementary Figure 5: D1R-MSNs shShank3 downregulated genes association with SFARI genes.**

**(a)** Differential expression analysis of AAV-scrShank3 vs AAV-ShShank3 shows small indirect transcriptional effect in non-infected samples, while infected samples display the stronger transcriptomic alterations. In both D1R+ **(b)** and D1R- **(c)** SFARI associated genes are altered, supporting the link between Shank3 downregulation with an autism-related phenotype. **(d)** Time spent in compartments for mice infected with scrShank3 or shShank3 and infused with vehicle or HC-067047 (paired-samples t-tests for object- vs. social-containing chambers: scrShank3 + veh *t*_(10)_ = 2.772, *p* = 0.020; shShank3 + veh *t*_(7)_ = 0.462, *p* = 0.658; shShank3 + HC-067047 *t*_(7)_ = 3.339, *p* = 0.012). **(e)** Distance moved during social preference test (one way ANOVA followed by Bonferroni’s multiple comparisons test: *F*_(3, 24)_ = 0.586, *p* = 0.630). Error bars report SEM.

**Supplementary Figure 6: *Shank3^+/-^* does not show social deficits in naïve conditions.**

**(a)** Left: representative western-blot images from NAc homogenate. Right: Optical density (OD) quantification (unpaired-samples t-tests between *Shank3^+/+^* and *Shank3^+/-^*: α-Shank3 *t*_(6)_ = 4.988, *p* = 0.002; β-Shank3 *t*_(6)_ = 0.178, *p* = 0.864; γ-Shank3 *t*_(6)_ = 1.493, *p* = 0.186). (**b**) Behavioral task paradigm. **(c, d)** Left: Time spent around the target during social preference test for *Shank3^+/+^* and *Shank3^+/-^* mice (paired-samples t-tests for object- vs. social: *Shank3^+/+^* *t*_(9)_ = 5.167, *p* < 0.001; *Shank3^+/-^* *t*_(12)_ = 3.026, *p* = 0.011). Right: juvenile preference index (one-sample t-tests against chance level = 0.5: *Shank3^+/+^* *t*_(9)_ = 5.617, *p* < 0.001; *Shank3^+/-^* *t*_(12)_ = 3.146, *p* = 0.008). **(e)** Time spent in the juvenile, object or in center chamber during social preference test for *Shank3^+/+^* and *Shank3^+/-^* mice (paired-samples t-tests for object- vs. social-containing chambers: *Shank3^+/+^* *t*_(9)_ = 3.269, *p* = 0.001; *Shank3^+/-^* *t*_(12)_ = 2.705, *p* = 0.019). **(f)** Distance moved during social preference test (unpaired-samples t-tests: *t*_(21)_ = 0.9307, *p* = 0.363).

**Supplementary Figure 7: Supplementary data on behavioral and electrophysiological experiments presented in Figure 4.**

LPS challenge induces social deficits in *Shank3^+/-^* mice after 24 hours: **(a)** Time spent in the juvenile, object or in the center chamber during social preference test for *Shank3^+/+^* and *Shank3^+/-^* previously injected with vehicle or LPS (paired-samples t-tests for object- vs. social-containing chambers: *Shank3^+/+^* + veh *t*_(7)_ = 4.838, *p* = 0.002; *Shank3^+/+^* + LPS *t*_(8)_ = 3.87, *p* = 0.005; *Shank3^+/-^* + veh *t*_(8)_ = 4.526, *p* = 0.002; *Shank3^+/-^* + LPS *t*_(9)_ = 0.3939, *p* = 0.703). **(b)** Distance moved during social preference test (two-way ANOVA followed by Bonferroni’s multiple comparisons test: LPS treatment main effect *F_(1,32)_ = 58.03, p <* 0.001). 7 days after LPS challenge, sociability and distance moved are not impaired anymore: **(c)** Time spent in the juvenile, object or in the center chamber during social preference test for *Shank3^+/+^* and *Shank3^+/-^* previously injected LPS (paired-samples t-tests for object- vs. social-containing chambers: *Shank3^+/+^* + LPS *t*_(7)_ = 5.083, *p* = 0.002; *Shank3^+/-^* + LPS *t*_(10)_ = 2.536, *p* = 0.032. **(d)** Distance moved during social preference test (unpaired t-test t*_(15)_* = 0.3116, p = 0.76). **(e)** Left: Example traces from 300 pA depolarizing current injection in putative D2 (pD2R) MSNs of *Shank3^+/+^* or *Shank3^+/-^* mice 24 hours after vehicle IP injection or LPS challenge. Right: Number of action potentials (nAPs) across increasing depolarizing current steps (0-400 pA) for D1R-tom-::*Shank3^+/+^*  or *Shank3^+/-^* MSNs (pD2R MSNs) after vehicle injection or LPS challenge (Two-way repeated measures ANOVA, treatment main effect *F_(2, 36)_ =* 0.030, *p* = 0.972, current steps main effect *F_(1.826, 65.75))_ =* 13.73, *p <* 0.001, treatment by current steps interaction *F_(16, 288)_ =* 0.1244, *p >* 0.999, from 3 mice each group). **(f)** Left: Example traces from 300 pA depolarizing current injection in D1R+ MSNs of *Shank3^+/-^* mice 24 hours or 7 days after vehicle IP injection or LPS challenge. Right: Number of action potentials (nAPs) across increasing depolarizing current steps (0-400 pA) for D1R-tom+::*Shank3^+/-^* MSNs 24 hours or 7 days after vehicle IP injection or LPS challenge (Two-way repeated measures ANOVA, treatment main effect *F_(2, 40)_ =* 3.829, *p* = 0.030, current steps main effect *F_(1.439, 57.56)_ =* 28.24, *p <* 0.001, treatment by current steps interaction *F_(16 , 320)_ =* 2.607, *p <* 0.001, from 3 mice each group). Error bars report SEM.

**Supplementary Figure 8: Overexpression of Trpv4 in the NAc during adulthood does not alter sociability and excitability of D1R+ MSNs in *Shank3*^+/-^ mice.**

**(a)** Schema of injection sites in the NAc with AAV-hSyn-eYFP or AAV-hSyn-mTRPV4-2A-eGFP in adult *Shank3*^+/+^ and *Shank3*^+/-^ mice. **(b)** Real-time PCR analysis of NAc dissected from adult *Shank3*^+/+^ and/or *Shank3*^+/-^ injected mice confirms the upregulation of *Trpv4* (unpaired t-test: *t*_(8)_ = 22.39, *p* < 0.001)*.* **(c, d, e** and **f)** Left: time spent around the enclosures during the social preference test for mice injected with AAV-hSyn-eYFP or AAV-hSyn-mTRPV4-2A-eGFP (paired-samples t-tests for object- vs. social: (c) *t*_(10)_ = 3.943, *p* = 0.003; (d) *t*_(10)_ = 3.626, *p* = 0.005; (e) *t*_(14)_ = 2.521, *p* = 0.024. Wilcoxon test for object- vs. social: (f) W = -45.00, *p* = 0.004). Right: juvenile preference index for mice injected with AAV-hSyn-eYFP or AAV-hSyn-mTRPV4-2A-eGFP (one-sample t-tests against chance level = 0.5: (c) *t*_(10)_ = 3.715, *p* = 0.004; (d) *t*_(10)_ = 4.068, *p* = 0.002; (e) *t*_(14)_ = 2.303, *p* = 0.037; (f) *t*_(8)_ = 3.537, *p* = 0.008). **(g)** Time spent in compartments for mice injected with AAV-hSyn-eYFP or AAV-hSyn-mTRPV4-2A-eGFP (paired-samples t-tests for object- vs. social-containing chambers: *Shank3*^+/+^ - eYFP *t*_(10)_ = 3.212, *p* = 0.009; *Shank3*^+/+^ - Trpv4 *t*_(14)_ = 2.974, *p* = 0.010; *Shank3*^+/-^ - Trpv4 *t*_(8)_ = 4.495, *p* = 0.002. Wilcoxon test: *Shank3*^+/-^ - eYFP W = -66.00, *p* = 0.001). Error bars report SEM.

**Supplementary Figure 9: LPS challenge increases the density of cFos positive neurons in the NAc.**

**(a)** Experimental design. Adult *Shank3^+/-^/*Drd1a-dTomato mice were injected with vehicle or LPS and were sacrificed after 24 hrs. **(b)** cFos-immunostaining of the NAc of *Shank3^+/-^/*Drd1a-dTomato mice (scale bar: 100 µm). (c) Quantification of cFos positive cells in the NAc (two-way ANOVA, treatment main effect *F_(1, 4)_ =* 19.75, *p* = 0.011, D1R main effect *F_(1, 4)_ =* 35.43, *p =* 0.004, treatment by D1R interaction *F_(1, 4)_ =* 0.760, *p =* 0.432). Error bars report SEM.

**Supplementary Figure 10: Supplementary data on behavioral experiments presented in Figure 6.**

**(a)** Time spent in compartments for *Shank3^+/-^* mice after LPS challenge and with vehicle or HC-067047 infusion in the NAc (paired-samples t-tests for object- vs. social-containing chambers: *Shank3^+/-^* + veh *t*_(6)_ = 1.3229, *p* = 0.232; *Shank3^+/-^* + HC-067047 *t*_(6)_ = 1.17, *p* = 0.286). **(b)** Distance moved during social preference test (unpaired t-test: *t*_(12)_ = 0.3708, *p* = 0.717). Error bars report SEM

**Supplementary Table legends**

**Supplementary Table 1:** Count tables normalized to reads per million (RPM). Genes were filtered keeping only those with more than 10 RPM.

**Supplementary Table 2:** List of selected genes from Table 1 after the worst-case scenario threshold of 1.5. These data were used as input for the PCA analysis.

**Supplementary Table 3:** List of genes clustered by Euclidean distance after normalizing the rlog transformation of the RPM count tables. These data were used as input for the heatmap in Fig. 3e.
